# Multi‐Purpose Data Worth Assessment of a Surface Water‐Groundwater and Nitrogen Transport Model

**DOI:** 10.1111/gwat.13490

**Published:** 2025-05-06

**Authors:** Patrick Durney, Antoine Di Ciacca, Scott Wilson, Thomas Wöhling

**Affiliations:** ^1^ Lincoln Agritech Canterbury New Zealand; ^2^ Institute of Hydrology and Meteorology Technische Universität Dresden Saxony Germany

## Abstract

Understanding which hydrological data types provide the most valuable information for models is crucial, given the limitations of data availability. This study applies data worth analysis to evaluate the impact of various observation types on predictive uncertainty in a coupled SWAT‐MODFLOW‐RT3D model simulating water flows and nitrate transport in a small headwater catchment in New Zealand. We assessed the worth of continuous nitrate concentrations, in‐catchment flow measurements, and SkyTEM‐derived groundwater levels for predicting stream flow and in‐stream nitrate concentrations. Using PEST software for model calibration and linear uncertainty analysis, we determined the relative worth of different observation types. Results indicate that SkyTEM estimates of groundwater levels and continuously measured nitrate concentrations were particularly effective in reducing predictive uncertainty. This study highlights the value of integrating high‐resolution SkyTEM data into models to enhance prediction accuracy for groundwater levels, stream flow, and nitrate pollution. It also demonstrates nitrate's utility as an environmental tracer, refining our understanding of surface water–groundwater interactions and solute transport in the Piako Headwaters Catchment.

## Introduction

Understanding the impact of land use changes on water resources is crucial for policy and environmental stewardship (Anand et al. 2018). This understanding hinges on local‐scale data, the cost of which often limits our ability to comprehensively assess land use change impacts (McIntyre et al. 2014). Hydrological models offer an alternative, allowing assessment of past land use changes and prediction of future impacts (Wang and Chen 2021). For small watersheds, coupled surface water‐groundwater models can provide valuable insights into the complex interactions between land use changes and water resources. These models often integrate multiple components to simulate both water quantity and quality. However, such complexity can be accompanied by significant uncertainties, particularly when applied to heterogeneous landscapes (Wei et al. 2019; Wang and Chen 2021; Rafiei et al. 2022). Uncertainty analysis is crucial for assessing the risk associated with decisions that are based on model predictions (Delottier et al. 2017). Brunner et al. (2012) conducted an uncertainty assessment for integrated hydrological models, focusing on the worth of different data types in reducing parameter and predictive uncertainty. Their study, which used both linear and nonlinear methods, provides valuable insights into the relative importance of various observation types for constraining vadose zone parameters and predictions. For example, they found that hydraulic head observations were most informative for future water table predictions, while evapotranspiration and soil moisture data had varying worth depending on the depth to groundwater. Building on this work, recent studies have continued to address uncertainty analysis in coupled models across various platforms. For example, Abbas et al. (2024) developed a framework for parameter estimation, sensitivity analysis, and uncertainty analysis for hydrological modeling using SWAT+. In the realm of fully integrated models, Srivastava et al. (2014) conducted a global sensitivity analysis using the Morris method on a ParFlow‐CLM model application to assess the spatially and temporally variable sensitivity of hydrological processes in a large, complex river basin, while Schilling et al. (2022) applied uncertainty analysis and data worth analysis to a coupled surface‐subsurface model using HydroGeoSphere. This research builds upon these studies assessing the relative value of different data types in reducing predictive uncertainty for coupled models, especially in small headwater catchments. Given the cost and time required for data collection, it is crucial to understand which data sources provide the most value in reducing model uncertainties. This field of investigation, known as data worth assessment (Gosses and Wöhling 2021; Wang et al. 2023), can help guide efficient monitoring strategies for small watersheds (Wöhling et al. 2016). One widely adopted approach for assessing data worth is the First‐Order Second‐Moment (FOSM) method, which is part of the PEST optimization and uncertainty software (Dausman et al. 2010; Doherty 2015, 2021; Partington et al. 2020; Schilling et al. 2022). FOSM is based on the sensitivity of model predictions to changes in input parameters and can identify the most important data to measure in order to reduce uncertainty in model predictions (Doherty 2015). We also apply FOSM techniques to evaluate the relative value of catchment outlet and in‐catchment monitoring data using a SWAT‐MODFLOW‐RT3D model in a small headwater catchment. Our work extends previous research by:

1. Applying data worth analysis to the SWAT‐MODFLOW‐RT3D coupled model, which is increasingly used for integrated water resource management.
2. Exploring the value of continuous nitrate measurements as a tracer for quantifying both nitrate and water transfer between surface and groundwater.
3. Evaluating the potential of a novel data type, namely airborne electrical resistivity (SkyTEM) derived groundwater levels, in reducing model uncertainty.
4. Conducting a spatial analysis of data worth across the catchment.

By demonstrating the application of data worth assessment in a small watershed model, our study provides practical insights for researchers and water resource managers working in similar contexts. The findings can help guide future monitoring efforts, potentially reducing costs and improving the efficiency of data collection strategies. Ultimately, this work contributes to enhancing our ability to model and understand the impacts of land use changes on local water resources, supporting more informed decision‐making in watershed management.

## Methods

### Study Area and Conceptual Model

#### Overview and Physical Setting

**Figure 1:**
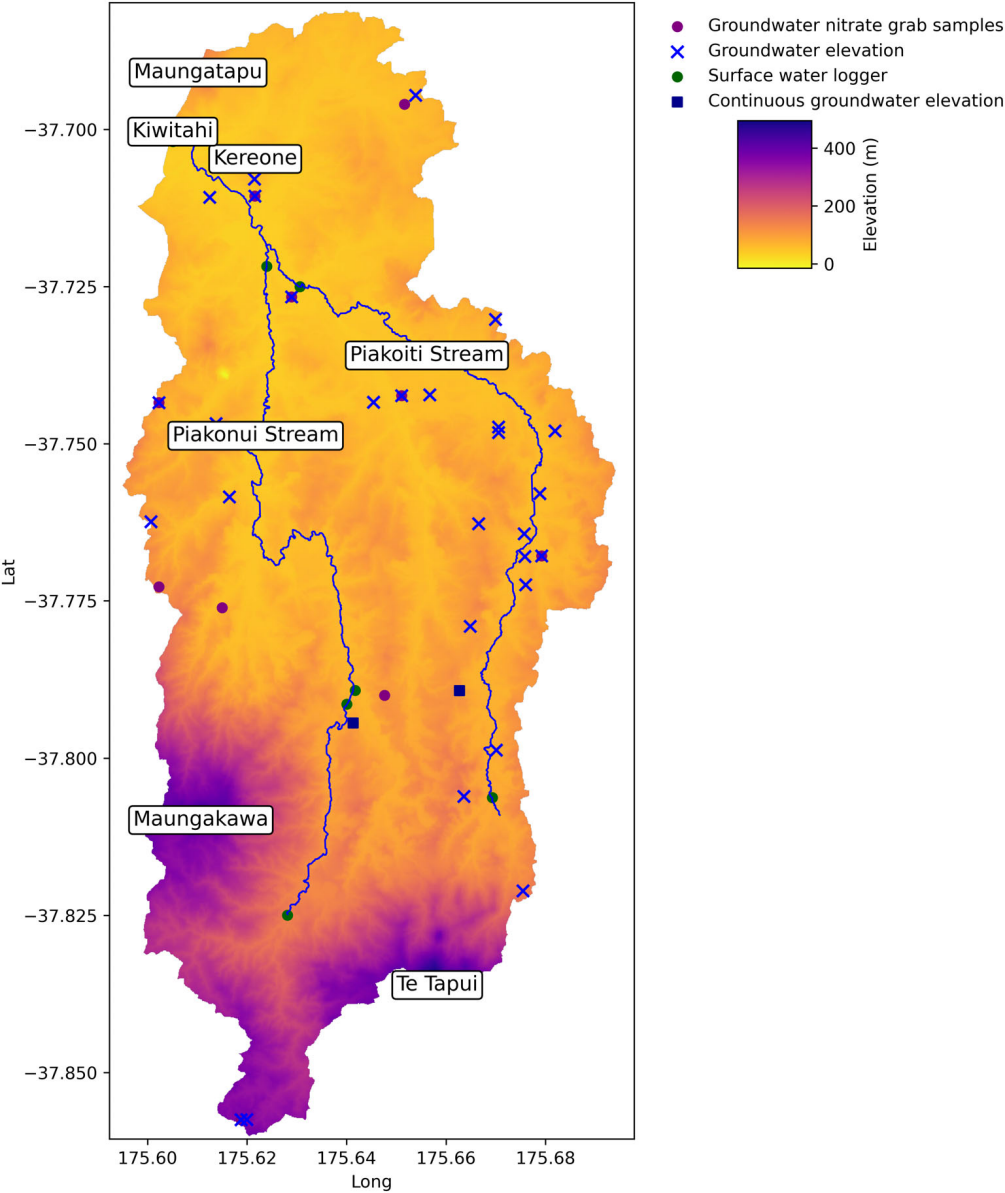
Study area and monitoring sites.

The Piako Headwater catchment is a small (108 km^2^) headwater catchment located in the Waikato Region of New Zealand (Figure 1). The landscape comprises rolling hills, with approximately 93.5 km^2^ used for dairy grazing and the remainder in native forest. Two main tributaries, the Piakonui and Piakoiti, originate on the Maungakawa and Te Tapui hills, respectively, at elevations of around 495–492 m. These tributaries merge near Kereone to form the Piako River, which exits the catchment at Kiwitahi (approximately 28 m elevation).

The catchment's geology is predominantly ignimbrites and andesitic basalt domes of the Kiwitahi volcanic group, with additional greywacke at higher elevations forming the geological basement (Healy et al. 1964). The ignimbrites vary from highly weathered material to relatively unaltered welded units (Leonard et al. 2010). Boreholes drilled for this study revealed a weathered layer extending to approximately 11 m, indicating a heterogeneous shallow subsurface.

SkyTEM electromagnetic aerial geophysical surveys were flown to improve geological classification of the study area. Data processing via Clara k‐medoids clustering (Jin and Han 2011) grouped the catchment into three principal hydrogeophysical units: welded ignimbrite, unwelded ignimbrite, and andesitic basalt. These units broadly align with historical geological mappings (Healy et al. 1964), though minor greywacke regions remain indistinct in the SkyTEM dataset (further details are provided in Appendix S1).

Woodward and Stenger (2018, 2020) identify that at the catchment outlet (Kiwitahi), more than 70% of total flow volume is estimated to come from groundwater, with 60% of the flow linked to rapid shallow groundwater pathways. The Piakonui tributary contributes 50–70% of flow at Kiwitahi and maintains continuous flow from its source, in contrast to the smaller Piakoiti, which intermittently dries. They identify that under high‐flow conditions, very shallow young groundwater and near‐surface runoff dominate, while low flows reflect deeper, older groundwater.

Agricultural pollution has degraded water quality throughout the catchment, with nitrate‐nitrogen (NO_3_–N) concentrations peaking around 5 mg/L during high‐flow events, compared to a baseflow background of about 0.5 mg/L. Groundwater yields are generally low (on the order of 10^−1^ to 10^0^ m^3^ day^−1^), with transmissivities usually less than 1 m^2^ day^−1^ (Waikato Regional Council 2022).

We propose that the rapid shallow groundwater flow pathway proposed by Woodward and Stenger (2018) derives from a weathered layer within the ignimbrite, which intercepts recharge and facilitates quick lateral movement. This behavior complements the standard soil zone interflow typically accounted for in models like SWAT. The presence of multiple interflow pathways in hilly terrain is consistent with Brantley et al. (2017).

Accordingly, we adopt a conceptual model with three flow reservoirs: a near‐surface reservoir encompassing surface runoff and vadose zone processes (as represented by SWAT's stream network and interflow components); a shallow groundwater reservoir within the uppermost 10 m, bounded by the weathered contact; and a deeper groundwater reservoir at unknown but significantly greater depths, carrying older and slower‐flowing groundwater.

### Data Sources and Measurements

#### Meteorological Data

Rainfall in the Piako Headwater region ranges between 1000 and 1400 mm year^−1^, predominantly falling in winter months (Chappel 2016), while Penman‐Monteith reference evapotranspiration is approximately 950 mm year^−1^. This study employs a daily, spatially variable climate dataset generated via random‐forest spatio‐temporal interpolation of long‐term regional records and four stations active from 2020 to 2022. Models were developed in *R* using the *mice* and *caret* packages (Kuhn 2008; van Buuren and Groothuis‐Oudshoorn 2011) to produce daily outputs for 2001 to 2022 over any (*x*, *y*, *z*) location in the catchment (Durney 2022).

#### Hydrological, Hydrogeological, and Water Quality Data

Waikato Regional Council maintains a 40‐year flow‐rated stage recorder at the Kiwitahi outlet (Figure 1) and two long‐term water quality sites in the catchment (monthly grab samples at Kiwitahi and Piakonui Rd) (Waikato Regional Council 2023a, 2023c). An additional five stage recorders have logged approximately 1.5 years of data. Two continuous nitrate monitoring sites (TriOS NICO sensors) on the Piakonui and Piakoiti tributaries offer measurements at 15‐min intervals, cleaned by removing invalid entries, filtering low‐quality data, and applying a density‐based Local Outlier Factor. Following cleaning, the data were resampled to daily averages; however, high‐flow nitrate values may still be underestimated due to turbidity issues.

Groundwater data include historical water‐level records for 29 bores, two newer continuously monitored sites, and a set of 12 one‐time water quality samples (Waikato Regional Council 2022, 2023b, 2023c). SkyTEM aerial geophysical data, from February 2019, add roughly 3776 estimates of groundwater table elevation (SkyTEM Aerial Geophysics Derived Groundwater Levels, SAGTDGWL). The methodology for their generation calculates mean resistivity at each sounding, identifying depth intervals where resistivity notably decreases, presumably indicating the transition from unsaturated to saturated conditions (Vang et al. 2023). Full details are provided in Appendix S3. These estimates were assigned to the model grid using a *k*‐nearest neighbors approach. Although uncertainties can exceed 5 m in some locations, most comparisons against known well data fell within acceptable confidence limits, enhancing understanding of the spatial variability in groundwater elevations during the SkyTEM flights.

Non‐SAGTDGWL monitoring sites appear in Figure 1, while SAGTDGWL points are omitted for clarity. Table 1 summarizes the full suite of monitoring data, including types, sources, station IDs, temporal coverage, measurement frequencies, and associated methods or equipment.

**Table 1:** Summary of Hydrological and Water Quality Data Used in This Study.

Data Type	Station Count	Temporal Coverage	Frequency	Method and Equipment
Flow	1^1^	40+ years	Continuous	Flow‐rated stage recorder
Flow	5^2^	1.5 years	Continuous	Stage recorders
Water quality	2^1^	Monthly	–	Chemistry grab samples
Nitrate	2^2^	1.5 years	15‐min intervals, daily avg	TriOS NICO sensors (10 mm path length, 0.05–6 mg/L NO_3_–N)
Groundwater levels	29^1^	Unknown	Spot	Dip meter
Groundwater levels	2^2^	2 years	15‐min intervals, daily avg	Pressure transducer
Groundwater quality	12^2^	One‐time samples	One‐time	Chemistry grab samples
SkyTEM derived Groundwater levels	3776^2^	February 18–19, 2018	One‐time	SkyTEM aerial geophysics, resistivity analysis

1 Data from Waikato Regional Council.

2 Data from this study.

### Numerical Model Overview

The 108 km^2^ study area was divided into 21 SWAT streams and sub‐basins derived from 1 m LiDAR data. Surface‐water modeling incorporated different land cover and soil types, with specific agricultural practices assigned to represent known land use. Daily runoff was estimated using SWAT's curve number approach, which depends on soil moisture, while potential evapotranspiration (PET) was computed via the Penman–Monteith method from a local climate station. Land cover was simplified into two categories: pastoral grazing and native forest.

Grazing management for dairy areas was informed by farm economic modeling, setting an initial daily dry mass consumption of 30 kg d^−1^ ha^−1^ and an effluent deposition of 12 kg d^−1^ ha^−1^. These parameters were incorporated in SWAT's land‐use management module. In the absence of fertilizer data, effluent deposition rates (with fixed scheduling) were adjusted during calibration to match observed NO_3_–N levels in ground and surface waters.

Groundwater was simulated in a coupled MODFLOW–SWAT setup. The MODFLOW model construction and calibration centered on the catchment geology, represented by two numerical layers to distinguish shallow and deep groundwater flow systems and to capture unique geological flow characteristics.

Given the catchment's complex geology, a pragmatic approach was adopted. Rather than attempting detailed stratification, materials were aggregated based on the SkyTEM clustering process. These simplified hydrostratigraphic units were then assigned to each model cell according to the modal (most common) SkyTEM‐derived classification in each numerical layer. Three categories were used: unwelded ignimbrite, welded ignimbrite, and basement materials.

A uniform vertical hydrogeology was assumed, as due to periodically unsaturated conditions in the upper model layer, separating hydrostratigraphic units in that layer was impractical. This approach allows a focused analysis of how different data streams, within a simplified geological framework, influence model performance. Further details on the clustering procedure can be found in Appendix S1.

Initial conditions for the coupled model were established through a multistep process, incorporating both uncoupled and coupled long‐term simulations to achieve equilibrium—particularly for the deeper groundwater layer (Appendix S2). An overview of the model components and data sources is presented in Table 2.

**Table 2:** Model Components and Data Sources Used in This Study.

Component	Details	Source	Resolution/Scale
Study area	107.607 km^2^	Calculated	N/A
Simulation period	7 years	N/A	N/A
Topography	LiDAR‐derived	Aerial Surveys 2018	1 m
SWAT subbasins	21 (avg. 5 km^2^ each)	Derived from LiDAR and monitoring locations	N/A
Hydrologic response units (HRUs)	113 total. Mean: 0.95 km^2^, Min: 0.01 km^2^, Max: 9.7 km^2^, Median: 0.26 km^2^	Derived from soil classes and land cover	N/A
Soil spatial data	6 soil types. Aggregated to a single 1 m thick layer in SWAT.	NZ fundamental soils layer and SMAP	1:50,000
Land cover	2 classes (Forest and Pasture)	Land Cover Database version 5 (MFE 2020)	1:50,000
MODFLOW grid	198 rows × 87 columns × 2 layers	Derived from resampled LiDAR	100 m × 100 m cells
Climate inputs	Subbasin level	RF and GBM model	N/A

The SWAT–MODFLOW–RT3D framework follows Wei et al. (2019). Within this setup, RT3D models NO_3_–N transport using a simplified reaction package. Dispersivity parameters were held constant, and a uniform reactivity parameter was applied throughout the model domain for computational efficiency. Porosity values were varied to capture heterogeneity in the study area.

Coupling SWAT and MODFLOW, including disaggregating SWAT's hydrological response units to the MODFLOW grid, was performed as detailed by Bailey et al. (2016) and Wei et al. (2019). Briefly, SWAT handles surface processes and soil profile nitrate transformations (plant uptake, nitrification, denitrification), passing any nitrate leaching below the root zone to MODFLOW as a concentration in recharge. RT3D then receives groundwater fluxes and NO_3_–N inputs from MODFLOW along with the SWAT‐derived recharge. It calculates groundwater NO_3_–N concentrations and loads to streams, sending these data back to SWAT for in‐stream water quality calculations. Additional details on model development and initial conditions can be found in Appendix S2.

**Figure 2:**
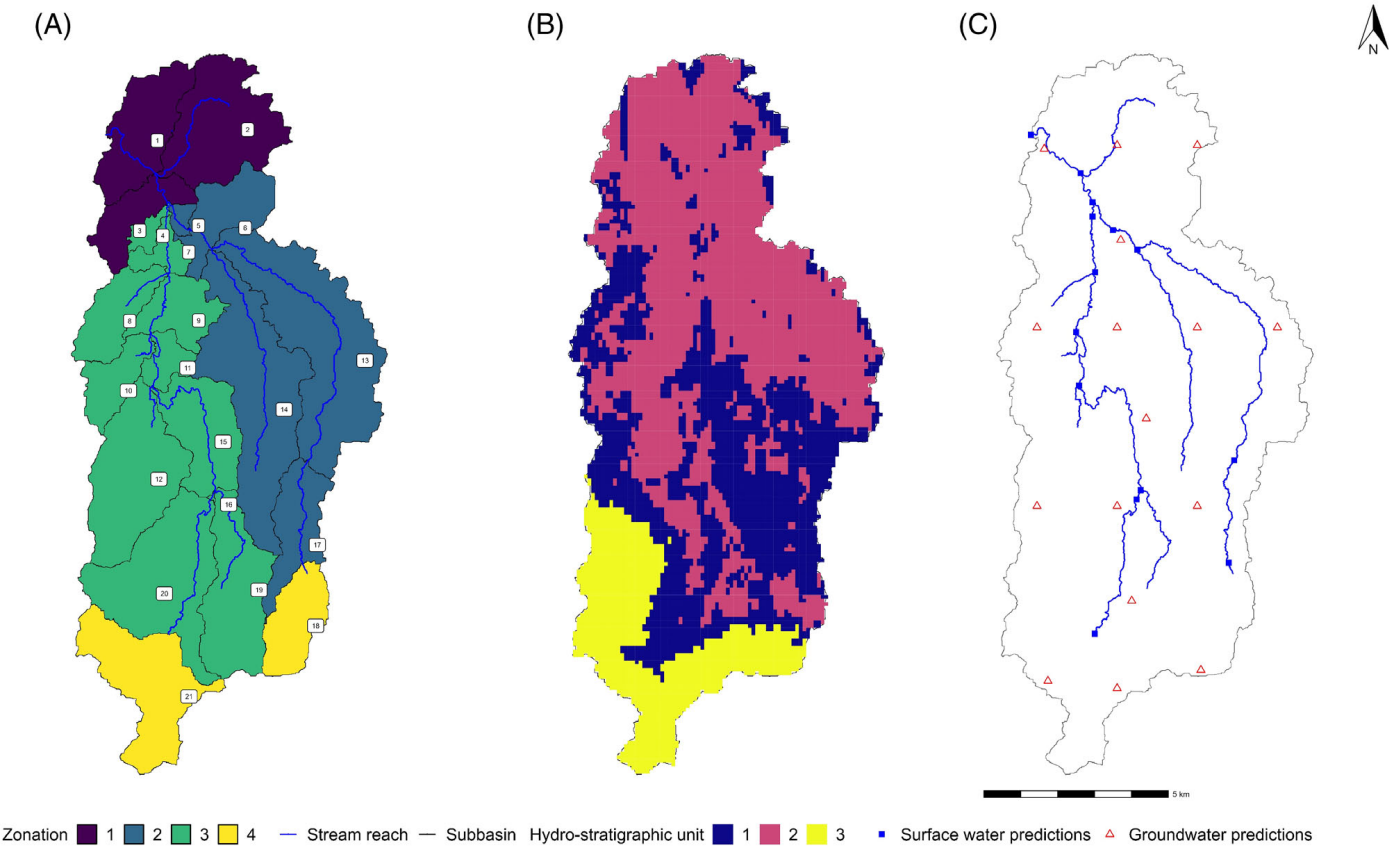
Zonation of SWAT parameters (a), hydrostratigraphic units (b), and data worth prediction sites (c).

Model parameters selected for calibration (Table 3) were identified through a multistep sensitivity analysis. They encompass both surface (e.g., Manning's roughness) and subsurface (e.g., hydraulic conductivity) properties, plus nutrient transport and transformation parameters (e.g., first‐order denitrification) and nutrient deposition rates. Parameter values were assigned to zones corresponding to distinct geographic or hydrogeophysical units, ensuring spatial variability was preserved. For instance, hydrogeophysical zones (Figure 2b) were derived from SkyTEM clustering to define uniform hydraulic properties, whereas SWAT parameters were divided by sub‐catchments (Figure 2a).

**Table 3:** Parameters Adjusted During Calibration.

Parameter Group	Parameter Name (Description)	A Priori Range	Posterior Range	Units
Groundwater flow	Kh (horizontal hydraulic conductivity for layer 1)	1^−4^ to 150	41.42, 76.42, 139	m/d
	Kh (horizontal hydraulic conductivity for layer 2)	1^−4^ to 20	1 × 10^−4^, 9 × 10^−4^, 0.0403	m/d
	Sy (specific yield for layer 1)	0.0001–0.2	1.3 × 10^−4^, 1.5 × 10^−4^, 1.5 × 10^−4^	—
	Sy (specific yield for layer 2)	0.005–0.4	5.6 × 10^−3^, 7.34 × 10^−3^, 0.315	—
	Vani (vertical anisotropy for layer 1)	1–20	1, 5.64, 13	—
	Vani (vertical anisotropy for layer 2)	0.1–20	0.214, 1, 2.27	—
	Porosity (effective porosity)	0.01–0.5	0.016, 0.0398, 0.0341, 0.0438	—
Surface water	MODFLOW river conductance (vertical conductance times length and width of stream segment in MODFLOW cell)	0.05–20,000	40, 53, 453, 6950	m^2^/d
	Manning's “n” (roughness coefficient for main channel and overland flow)	0.025–0.1	0.028–0.05	—
	Baseflow alpha factor (for main channel and bank storage)	0.05–1	0.09–0.547	1/d
	Groundwater delay (time for water leaving the bottom of the root zone to reach the shallow aquifer)	0.05–100	0.2–27.4	d
	Average slope length (for sheet flow and lateral subsurface flow)	0.1–200	2.4–104	m
Soil properties	Soil available water content (amount of water available to plants when the soil is at field capacity)	60–250	200–243	mm
	Soil ksat (saturated hydraulic conductivity)	2–180	2.77, 105, 128	cm/h
Nutrient dynamics	First‐order rate constant for denitrification (rate of denitrification)	0.0001–10	4.88 × 10^−02^	1/d
	Monod half‐saturation term for denitrification (substrate concentration at which the reaction rate is half of the maximum)	0.1–600	34.745	mg N/L
	Half‐life of nitrate in shallow aquifer (time for half of the nitrate to degrade in the shallow aquifer)	1–1000	16.1	d
	Nitrate percolation coefficient (fraction of nitrate that moves with water from one layer to the underlying layer)	1 × 10^−4^ to 1	0.00583	—
	Denitrification exponential rate coefficient (controls the rate of denitrification)	0.01–0.99	0.217	—
	Denitrification threshold water content (water content above which denitrification occurs)	0.01–2	0.9507	mm H_2_O
SWAT‐specific	Surface runoff lag coefficient (portion of surface runoff held in surface storage)	1–40	16.6	—
	Canopy interception (pasture)	0.1–4	1.43	mm
	Canopy interception (native forest)	0.5–10	0.86	mm
	Pasture dry mass consumption (amount of dry biomass consumed by grazing)	20–40	27–40	kg/ha
	Effluent dry mass (amount of dry biomass in effluent)	8–15	10–12	kg/ha

### Model Calibration, Uncertainty, and Data Worth

The goal of model calibration is to adjust model parameters to minimize the difference between model outputs (*o*) and corresponding observations (*h*). This requires the specification of an objective function (Φ) such as the summed square of residuals (*r*) between *o* and *h* used in this study. This study used the inverse of observation error to weight the observations during the calibration process. The weighted squared difference between model simulations and observations can be mathematically expressed as:

(1) $$\Phi ={\left(h-\mathrm{Xp}\right)}^{T}Q\left(h-\mathrm{Xp}\right)$$

where *X* is the sensitivity (Jacobian) matrix, *p* is the estimated parameter vector, and *Q* is the weighting factor matrix. Note here *T* represents the matrix transpose not a variable. This expression serves as the objective function for the calibration process which used BeoPEST, a parallel version of PEST (Doherty et al. 2010; Doherty 2015; Wöhling et al. 2016).

In our approach, we employed full‐record calibration rather than traditional split‐sample validation as we are investigating a value of the observations used during calibration. This decision acknowledges findings by Arsenault et al. (2018), who demonstrated that using the full dataset for calibration can yield more robust parameter sets and superior model accuracy across multiple hydrological models and catchments. Given purpose of the study and our limited data period, we used all available data for calibration, spanning from January 1, 2017 to March 1, 2023. The concept of First Order Second Moment (FOSM) linear uncertainty has been widely employed in prior groundwater‐only studies and is implemented here using PEST tools GENLINPRED and PREDUNC (Christensen and Doherty 2008; Dausman et al. 2010; Doherty et al. 2010; Doherty 2015, 2021; Zell et al. 2018) In this context, a particular model prediction (*s*) can be described by:

(2) $$s=y^{T}p$$

where *y* is the sensitivity of the prediction to the parameter vector (*p*). This equation enables the derivation of the expression for the prediction uncertainty variance (*σ*_s_) for a given *s* as:

(3) $$\sigma_{s}^{2}=y^{T}C_{p}y-y^{T}C_{p}X{\left(XC_{p}X^{T}+\frac{\Phi }{\left(n-m\right)Q}\right)}^{-1}XC_{p}y$$

where *C*_*p*_ is the innate covariance matrix of parameters, $\frac{\Phi }{\left(n-m\right)Q}$ is the reference variance, *n* is the number of observations and *m* is the number of estimated parameters.

In Equation 3, *y* ^*T*^ *C*_*p*_ *y* represents the prior uncertainty, while $y^{T}C_{p}X{\left(XC_{p}X^{T}+\frac{\Phi }{\left(n-m\right)Q}\right)}^{-1}XC_{p}y$ describes the reduction in prior uncertainty by a given data set (Dausman et al. 2010; Wöhling et al. 2016).

While a calibrated model can serve as a useful starting point for FOSM analysis, as in our study, enabling more realistic accounting for parameter sensitivities, it is important to note that FOSM primarily relies on the sensitivity of model outputs to parameters and observations, rather than on the absolute accuracy of model predictions (Brunner et al. 2012). The technique can be applied with initial parameter estimates that allow for the computation of these sensitivities, without necessitating a fully optimized calibration (Dausman et al. 2010). As Wallis et al. (2014) demonstrate, the focus of FOSM data worth analysis is on the relative value of different data types in reducing prediction uncertainty, rather than on achieving a perfectly calibrated model. In our study, we use this equation as an expression of data worth of a wide range of observation types for different model predictions. We used two methods to evaluate data worth in this study first detailed in (Dausman et al. 2010). Firstly, we use the decrease in the prior uncertainty variance (Method 1). This is calculated with each observation group notionally set as the only calibration constraint. The decrease in prior prediction uncertainty variance indicates the ability to constrain model uncertainty with a single observation group. Secondly, we use the increase in posterior uncertainty variance (Method 2). This is calculated by iteratively omitting each observation group from the calibration dataset. The degradation rate gives the model predictive ability without that particular observation group included in the calibration dataset, thereby providing a measure of the uniqueness of the information contained within that observation group.

We have examined the worth of the data used in calibration for informing predictions corresponding to the 5th, 50th, and 95th percentiles of stream flow and in‐stream NO_3_–N concentrations at all 21 SWAT sub‐basin outlets and groundwater levels and NO_3_–N concentrations at 16 approximately evenly distributed locations across the MODFLOW model domain (Figure 2c): This percentile‐based approach captures a range of environmental conditions (low, median, and high) across the modeled time series, allowing evaluation of data worth under varying hydrological and water quality conditions. The 5th percentile represents low‐flow, groundwater level or concentration conditions, the 50th percentile reflects typical (median) conditions, and the 95th percentile captures high‐flow, groundwater level or concentration events which may be relevant for contaminant transport studies. The quantile approach enables a comprehensive assessment of data worth across different conditions without the computational burden of analyzing every time step, while accounting for spatial and temporal variability in both surface water and groundwater systems. In addition, we assessed the data worth of observations for predicting flow partitioning for the total domain (volumetric discharge via surface run‐off and lateral flow versus shallow and deep groundwater).

## Results

### Overview of Model Calibration and Model Result Consistency with Prior Research

**Figure 3:**
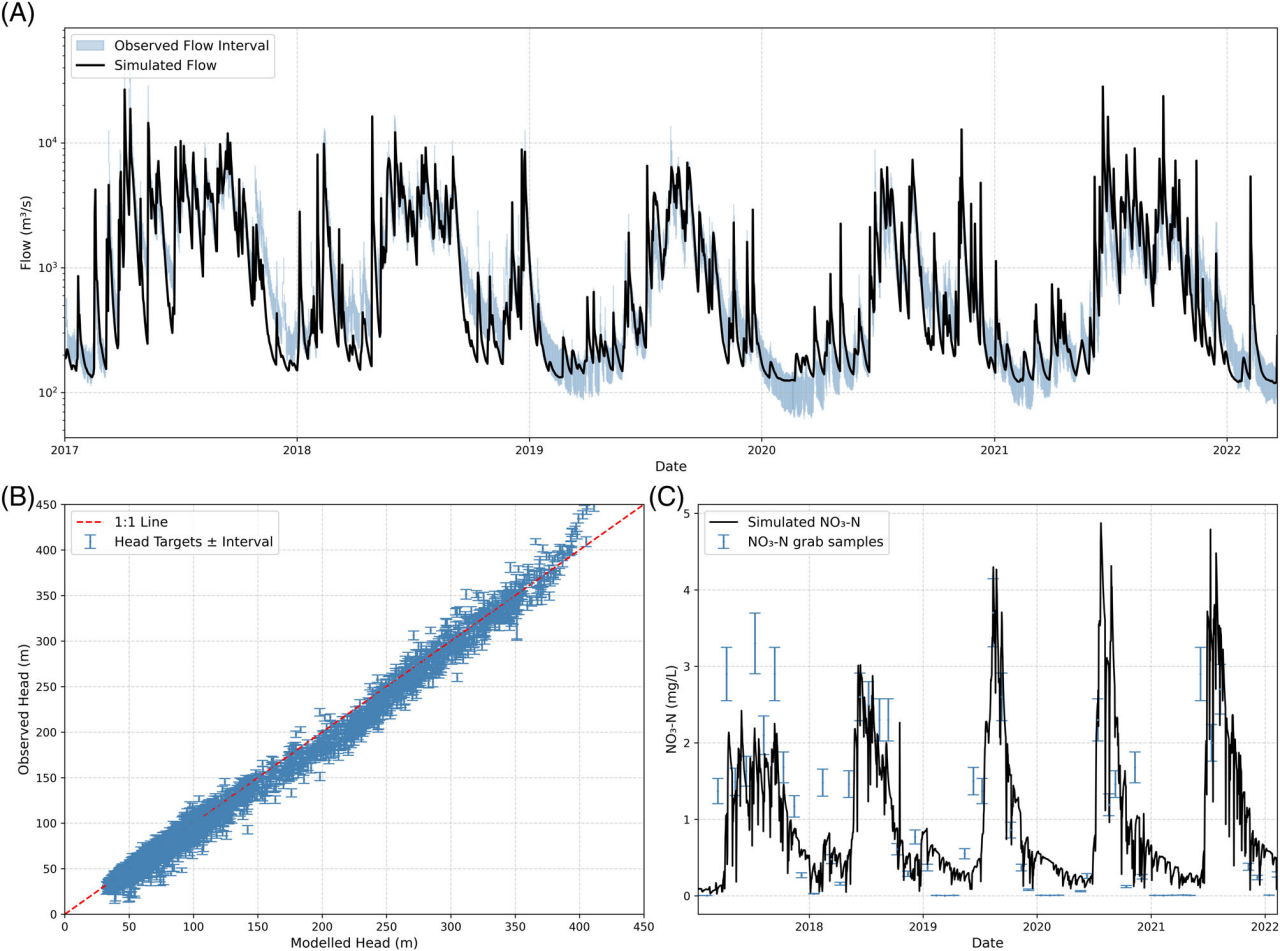
Example of calibration to flow at Kiwitahi (a), SkyTEM estimates of groundwater levels (b) and NO_3_–N at Kiwitahi (c), where steel blue interval represents the observation uncertainty and the black line represents the model prediction.

The main focus of this work is data worth assessment rather than model optimization; however, we provide a brief summary of the optimization with more detailed information available in Appendix S2. We evaluated the model's ability to simulate stream flows, groundwater levels, and nitrate concentrations for both surface and groundwater components. Table 4 summarizes the performance metrics. Figure 3a compares the modeled and observed streamflow at Kiwitahi, with the shaded area indicating observation uncertainty. Figure 3b presents modeled versus SkyTEM‐derived groundwater levels across the catchment. Figure 3c displays the modeled versus observed nitrate concentrations at Kiwitahi, with the shaded area representing observation uncertainty. Model calibration and performance evaluation were conducted with a primary focus on observational uncertainty, which is substantial in our study area. For instance, the 95% confidence interval for mean flow at Kiwitahi ranged from ±25% to ±85% of the mean flow between 1999 and 2022. Such significant uncertainties underscore the limitations of relying solely on standard performance metrics for model evaluation. Given the large uncertainty of our observations, assessing model performance against a singular observation value is unreasonable when actual values fall within a wider confidence interval. The uncertainty in our data means that traditional performance metrics, which do not account for observational uncertainties, could lead to biased parameters and unrealistic assessments of model performance. Therefore, we employed error‐normalized metrics in addition to standard metrics (which are presented in the Supporting Information). This is inspired by Bayesian theory and also aligns with the implementation in the suite of PEST calibration tools that weight observations by the inverse of their measurement error. This approach ensures that the model is informed in the calibration process considering the respective confidence in different data types.

**Table 4:** Calibration Metrics.

Component	Metric	Performance
Stream Flow	Error‐normalized NSE	Near unity at most sites, lower at PKT5
Surface Water NO_3_–N	Normalized PBIAS	Adequate at 3 of 4 sites
Groundwater Levels (STDGWL)	R^3^	>0.99
Groundwater Levels (STDGWL)	Error‐normalized RMSE	5.18 m
Groundwater Levels (One‐off)	Error‐normalized RMSE	9.7 m
Groundwater Levels (Transient)	Normalized RMSE	0.2 m
Groundwater NO_3_–N	R^3^	0.28
Groundwater NO_3_–N	Error‐normalized RMSE	1.48 mg/L

We also investigated parameter sensitivity, via identifiability analysis, during calibration, which revealed that the model is particularly sensitive to groundwater parameters such as hydraulic conductivity (Kh), specific yield (Sy), and vertical anisotropy and SWAT parameters governing recharge and nitrate load generation. Following calibration, these parameters showed the greatest reduction in uncertainty and largest levels of identifiability. In contrast, parameters with more localized effects, such as Manning's roughness, or those operating at sub‐daily timescales showed lower identifiability. This high sensitivity to groundwater and recharge parameters underscores the base flow‐dominant nature of the catchment's hydrograph.

### Predictive Uncertainty and Data Worth

Reduction in prior predictive uncertainty for each prediction type is presented in Table 5, which shows prediction uncertainty is significantly reduced for all observation types following calibration, including for the respective percentiles of each prediction type.

**Table 5:** Changes in Prediction Uncertainty Variance Following Model Calibration.

Prediction Group	Flow Percentile	Prior	Posterior	Reduction (%)
gw head (m)	5	7966.72	0.517	99.9
	50	8100.42	0.488	99.9
	95	7917.95	0.628	99.9
gw nitrate (mg/L)	5	0.0827	0.001	99
	50	3.1545	0.089	97.2
	95	9.0521	0.179	98
sw discharge (m^3^/d)	5	1042.69	2.11	99.8
	50	5311.21	48.81	99.08

**Figure 4:**
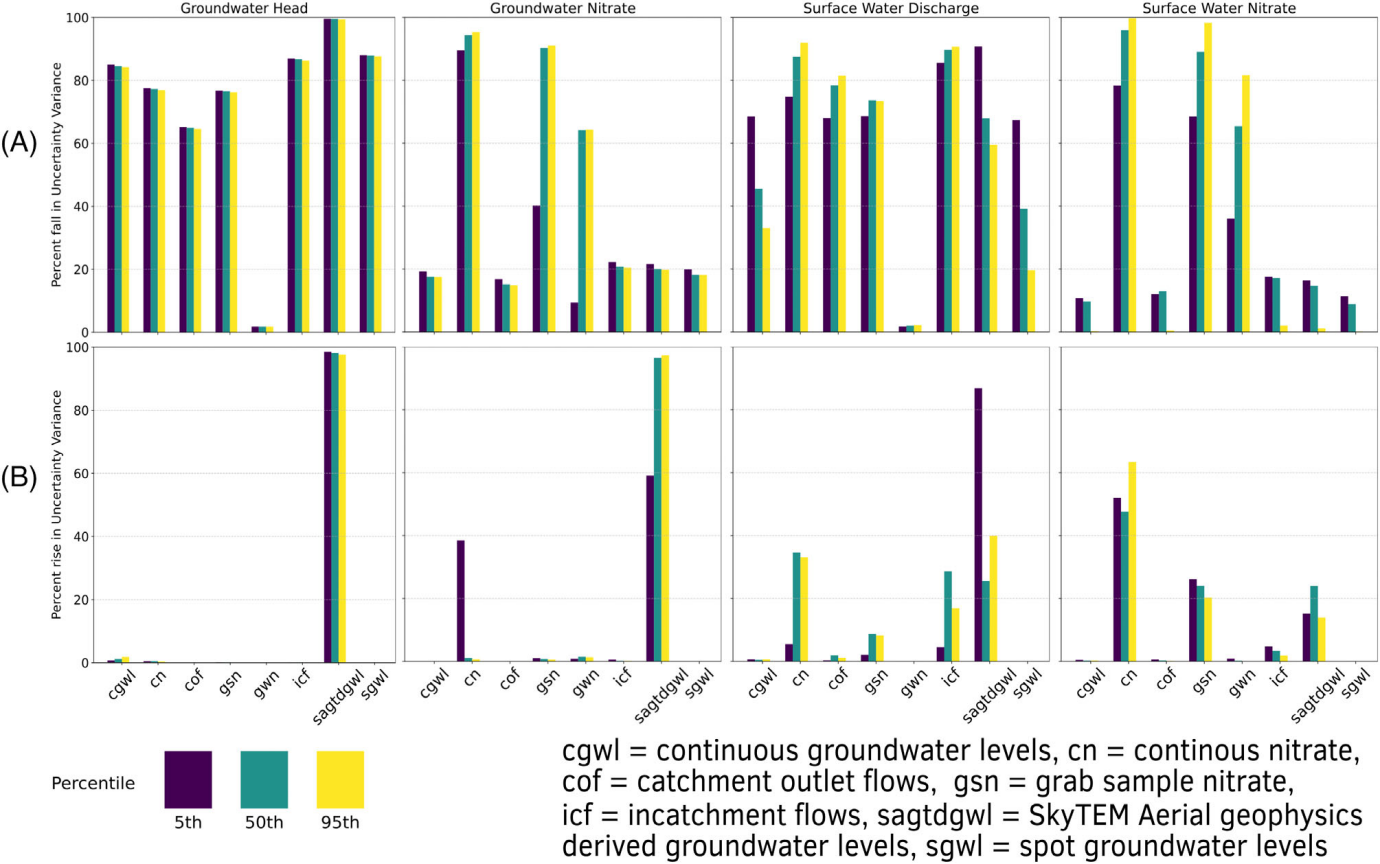
Changes in prediction uncertainty across the model domain. (a) How adding each type of observation alone reduces prior uncertainty. (b) How removing each type of observation increases posterior uncertainty while all other types remain.

Figure 4a and 4b display information about various observation groups across the entire model domain. Appendix S5 provides a relative ranking by observation group. Figure 4a quantifies each observation group's ability to reduce prior predictive uncertainty variance for each prediction type (method 1), while Figure 4b illustrates the effect of omitting each observation group on posterior uncertainty variance (method 2).

**Figure 5:**
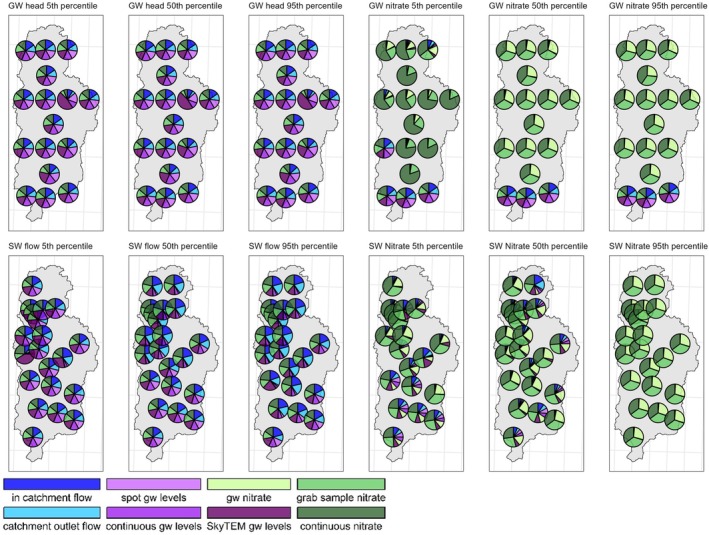
Relative contribution by fall in uncertainty variance accrued with the inclusion of each observation group as the sole member of the calibration dataset for groundwater sites.

**Figure 6:**
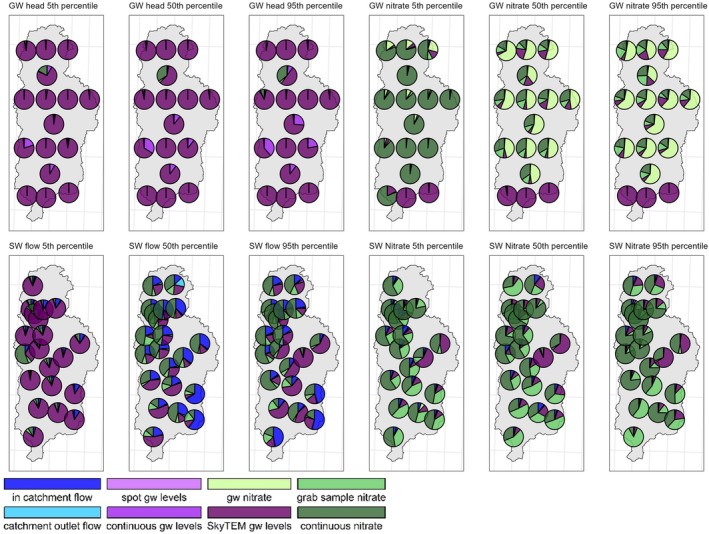
Relative contribution of each group to the relative rise in post‐calibration predictive uncertainty by the omission of each observation group from the calibration dataset.

Figure 5 illustrates the spatial variations in data worth according to Method 1. For water flows exceeding the 50th percentile, data related to surface water flows show increased relevance, particularly in the eastern and southern sub‐catchments of the modeling domain. The spatial patterns of data worth observed in Figure 5 generally persist across different locations. Figure 6 depicts the spatial variation in the rise of posterior uncertainty variance, as determined by Method 2. This visualization reveals heightened spatial variability in the uniqueness of datasets, particularly for groundwater nitrate concentrations and surface water discharge.

For groundwater nitrate predictions exceeding the 50th percentile, in the model's southern region, SAGTDGWL data is the most informative. In most other regions, discrete groundwater nitrate samples provide more unique information, while the absence of SAGTDGWL data leads to a marked rise in posterior uncertainty, primarily in the southern region.

In general for surface water discharge predictions, under low‐flow scenarios (below the 50th percentile), the lack of SAGTDGWL data has the most detrimental effect on model performance. During high‐flow conditions, the exclusion of continuous nitrate monitoring data leads to the most significant increase in posterior uncertainty. These effects vary longitudinally along the Piakonui and Piakoiti rivers; for instance, for the Piakoiti River in‐catchment flow measurements and SkyTEM water levels contain the most information. While the Piakonui River largely relies on continuous nitrate measurements in surface water.

For surface water nitrate concentrations across the model's domain, continuous nitrate monitoring contains the most information. In areas proximate to monthly grab samples, these data emerge as the most informative, while in the lower regions of the Piakoiti River, SAGTDGWL data contain the most information.

## Discussion

### Model Calibration

The calibration process revealed valuable insights into the catchment's hydrological behavior and the model's performance. Our approach, which incorporates both surface water and groundwater targets for flow and nitrate concentrations, highlighted the crucial role of groundwater processes in the overall system behavior. Parameter sensitivity analysis revealed the baseflow‐dominant nature of the catchment's hydrograph. The groundwater model's two‐layer structure, with higher conductivity in Layer 1 and lower in Layer 2, captured this behavior effectively. However, the models fit to groundwater level observations varied across the catchment, particularly in the upper Piakonui area, reflecting the complex interplay of local hydrological processes. Challenges emerged in accurately representing spot groundwater level observations, especially in steep terrain areas, potentially indicating structural biases. These difficulties underscore the complexity of modeling groundwater dynamics in heterogeneous landscapes and highlight the importance of considering topographic influences on groundwater flow. Additionally, limitations in simulating groundwater nitrate concentrations were observed, likely due to sparse observational data and structural aspects of the model. Despite these challenges, the calibrated parameter set and resulting water balance generally align with the conceptual understanding of the catchment's hydrology and previous Bayesian chemistry‐assisted hydrograph separation and load partitioning model (BACH) by Woodward and Stenger (2018, 2020) and Stenger et al. (2024). This alignment lends credibility to the model's overall representation of the catchment's hydrological processes. Our approach to model evaluation, which considers observational uncertainty, provides a continuity of narrative for subsequent data worth analysis and result interpretation. It acknowledges the variability in model accuracy across different sites and parameters, offering a comprehensive view of the model's strengths and limitations.

### Data Worth Analysis

The data worth analysis, illustrated by Figure 4a and 4b, provides compelling insights into the value and uniqueness of information within various observation groups across the entire model domain. The SAGTDGWL observation group stands out for its ability to reduce prior predictive uncertainty in groundwater levels, achieving >99% reduction in prior uncertainty variance. This underscores the potential of SkyTEM‐derived groundwater level data in constraining hydrological models. Spot measurements and continuous groundwater records also make valuable contributions, reducing prior uncertainty by 80–90%, although their impact is less pronounced than SAGTDGWL. These findings suggest that while traditional groundwater monitoring methods remain valuable, innovative techniques like SkyTEM can provide substantial improvements in model constraint, in particular in data‐sparse areas.

Continuous nitrate observations, despite having shorter records, outperform other nitrate observation groups in terms of information content. They provide insights into surface and groundwater nitrate dynamics and significantly inform predictions of surface water discharge. Their superior performance may be partially attributable to the lower measurement uncertainties associated with nitrate levels compared to flow targets, as observations were weighted by the inverse of their measurement uncertainty. The high value of continuous nitrate data emphasizes the importance of high‐frequency water quality monitoring in capturing the dynamic nature of nutrient transport in catchments.

Spatial variations in data worth, shown in Figures 4 and 5, highlight the model's variable behavior as a function of both geographic location and flow conditions, underscoring the complexity of the hydrological system. The importance of different data types varies under different flow conditions, revealing the dynamic information content in hydrological data. SAGTDGWL data is crucial during low‐flow conditions, likely reflecting the dominant role of groundwater in maintaining baseflow. Conversely, continuous nitrate monitoring is significant during high‐flow conditions, suggesting its value in capturing rapid changes in water quality and quantity during storm events or seasonal variations. This may be a result of high uncertainty in flow measurements relative to the nitrate concentrations during high‐flow conditions, perhaps due to insufficient gaugings. These findings emphasize the need for diverse data collection strategies that can capture the full range of hydrological conditions.

The contrasting data influences observed for the Piakoiti and Piakonui rivers further illustrate the importance of tailored monitoring approaches and that monitoring networks can be designed to optimize its value for (informing) catchment management. The intermittent nature of the Piakoiti River flow necessitates a more comprehensive understanding of flow dynamics and surface water–groundwater interactions, hence the importance of in‐catchment flow measurements and SAGTDGWL. In contrast, the continuous flow in the Piakonui River allows continuous nitrate measurements to serve as an effective integrator of both flow and surface water‐groundwater interaction. These differences highlight how local hydrological characteristics can significantly influence the most effective monitoring and modeling strategies. The site‐specific role of SAGTDGWL data in shaping nitrate prediction uncertainties in the lower regions of the Piakoiti River is particularly intriguing. One possible explanation is a strong groundwater influence on surface water nitrate concentrations in this area, potentially due to unique geological features or land‐use practices. It emphasizes the importance of considering local variations in groundwater‐surface water interactions when developing water quality models and management strategies. Moreover, it demonstrates how data worth analysis can reveal unexpected relationships and guide targeted investigations of local hydrological processes.

### Model Limitations and Future Directions

While the SWAT‐MODFLOW‐RT3D framework adopted in this study shows considerable promise, certain model simplifications may influence the reliability of data worth evaluations conducted using PEST and linear uncertainty analysis. For example, the simplified nutrient parameterization and uniform reactivity parameters in the RT3D module may limit the model's ability to capture fine‐scale spatial variability in nutrient transport and transformation processes. This simplification could affect the assessment of parameter uncertainty and the evaluation of data worth, particularly for observations related to water quality. Future efforts to incorporate greater spatial detail in nutrient and reactivity parameterization could enhance the robustness of data worth estimates, especially when integrating data from advanced sources such as aerial electromagnetic (AEM) measurements.

Similarly, sharp contrasts in the hydraulic properties between hydrogeological zones could lead to non‐linearities in model outputs that might bias the parameter sensitivity of the calibrated model and thus the relative worth of data. However, we believe that the effect of unknown (or unresolved) structural features in the subsurface is equally—if not more—important. The uncertainty analysis approach applied here is applicable for linear (or weakly non‐linear) models and is a computationally efficient method for models with large run‐times. If computationally feasible, our methods can be expanded easily to Bayesian techniques that do not rely on the linearity assumption (e.g., Leube et al. 2012).

## Summary

This study evaluated the relative worth of various data types in reducing predictive uncertainty variance within a SWAT‐MODFLOW‐RT3D model applied to a small headwater catchment in the Waikato region, New Zealand. The analysis utilizes continuous nitrate concentrations, in‐catchment flow measurements, and airborne geophysics derived groundwater level estimates (SAGTDGWL). Model calibration was performed using PEST software, followed by linear uncertainty estimation to assess parameter and prediction uncertainty and determine the relative worth of eight observation types.

The key findings highlighted the high data worth of continuous nitrate measurements and SAGTDGWL, which proved effective in reducing uncertainty for groundwater levels, stream flows, and in‐stream nitrate concentrations. Continuous nitrate data was particularly valuable for reducing uncertainty in both nitrate concentrations and stream flow predictions. However, high uncertainty in rated flows diminished their value, suggesting that reducing this uncertainty could enhance their relative worth. Despite limitations, such as single‐day SAGTDGWL observations and limited continuous nitrate monitoring points, in‐catchment observations were crucial in reducing predictive uncertainty across various hydrological parameters. The study shows the role continuous nitrate data can play as a tracer for water movement across the Piako Headwater catchment and demonstrated that integrating multiple data types significantly reduced predictive uncertainty for surface water flow, nitrate concentrations, and groundwater levels.

The findings emphasize the importance of using a diversified dataset tailored to specific predictions for improving model accuracy in interconnected groundwater and surface water systems. They contribute to the growing body of research using nitrate as a tracer to estimate groundwater residence times and ages. These insights may help guide the selection of observation types for evaluating the impacts of land and water resource management practices, providing a robust basis for policy decisions. Future research should further explore the potential of aerial electromagnetic data in hydrogeology, given its capability to provide high‐resolution imaging of subsurface.

## Supporting information


**Appendix S1.** Geology and hydrogeology.
**Appendix S2.** Model development and calibration.
**Appendix S3.** SkyTEM aerial geophysics derived groundwater levels.
**Appendix S4.** Flow target derivation.
**Appendix S5.** Relative ranking of data observation types.

## Data Availability

The data that support the findings of this study are available from the corresponding author upon reasonable request.
