# Supplementary material for: Multi‐Purpose Data Worth Assessment of a Surface Water‐Groundwater and Nitrogen Transport Model
Source: Ground Water. 2025 May 6;63(4):580–94. doi: 10.1111/gwat.13490 (PMC12271998; doi:10.1111/gwat.13490)
Supplement: Supplementary file 1 — Appendix S1. Geology and hydrogeology. Appendix S2. Model development and calibration. Appendix S3. SkyTEM aerial geophysics derived groundwater levels. Appendix S4. Flow target derivation. Appendix S5. Relative ranking of data observation types. [file GWAT-63-580-s001.docx]

# **Multi-purpose data worth assessment of a surface water-groundwater and nitrogen transport model – Supporting information**

Patrick Durney, Corresponding author: Lincoln Agritech Ltd, Engineering Drive, Lincoln University, Lincoln, Canterbury, New Zealand; Komanawa Solutions Ltd, Christchurch, Canterbury, New Zealand, patrick@komanawa.com

Antoine Di Ciacca, Lincoln Agritech Ltd, Engineering Drive, Lincoln University, Lincoln, Canterbury, New Zealand, antoine.diciacca@lincolnagritech.co.nz

Scott Wilson. Lincoln Agritech Ltd, Engineering Drive, Lincoln University, Lincoln, Canterbury, New Zealand, scott.wilson@lincolnagritech.co.nz

Thomas Wöhling. Lincoln Agritech Ltd, Engineering Drive, Lincoln University, Lincoln, Canterbury, New Zealand; Institute of Hydrology and Meteorology, Technische Universität Dresden, Dresden, Saxony, Germany, [thomas.woehling@tu-dresden.de](mailto:thomas.woehling@tu-dresden.de)

**Abstract**

Understanding which hydrological data types provide the most valuable information for models is crucial, given the limitations of data availability. This study applies data worth analysis to evaluate the impact of various observation types on predictive uncertainty in a coupled SWAT-MODFLOW-RT3D model simulating water flows and nitrate transport in a small headwater catchment in New Zealand. We assessed the worth of continuous nitrate concentrations, in-catchment flow measurements, and SkyTEM-derived groundwater levels for predicting stream flow and in-stream nitrate concentrations. Using PEST software for model calibration and linear uncertainty analysis, we determined the relative worth of different observation types. Results indicate that SkyTEM estimates of groundwater levels and continuously measured nitrate concentrations were particularly effective in reducing predictive uncertainty. This study highlights the value of integrating high-resolution SkyTEM data into models to enhance prediction accuracy for groundwater levels, stream flow, and nitrate pollution. It also demonstrates nitrate's utility as an environmental tracer, refining our understanding of surface water-groundwater interactions and solute transport in the Piako Headwaters Catchment.

# **Disclaimer**

Supporting Information is generally not peer reviewed.

# **Appendix S1 Geology and Hydrogeology**

This study aimed to refine the understanding of the geology of the study area through the utilisation of extensive electrical resistivity surveys conducted by SkyTEM Australia and inverted by Aarhus University. The surveys were flown in late February 2018 (21^st^ and 22^nd^) The method applied in this study is similar to that of Christensen et al. (2017). However, the availability of borehole logs for the study area was found to be limited, of low quality and unreliable, making the interpretation of resistivity data challenging.

To overcome these limitations, unsupervised machine learning algorithms were employed to cluster similar resistivity values based on both the sharp and smooth inversions and their physical location data. The k-medoids clustering algorithm was implemented using the *Clara* package in R to achieve this. *Clara* (clustering of large applications) uses a random sample of the input data to find a set of representative medoids and then uses these medoids to assign the remaining data points to clusters.

The results of the initial clustering attempts showed bias due to the use of three independent spatial parameters (Easting, Northing and elevation). To address this issue, the spatial information was transformed through a principal component analysis (PCA) to generate a single eigenvalue (dimensionality reduction). The eigenvalues were used in the clustering algorithm instead of the original spatial coordinates.

To facilitate the clustering process, the influence of water content on ground resistivity was taken into consideration by categorising the dataset into two distinct groups: saturated and unsaturated material. This demarcation postulated that the initial substantial negative deviation from the mean resistivity within a specific sounding delineated the shift from saturated to unsaturated conditions. However, upon analysis, it became apparent that the fixed depth intervals present in the inverted resistivity datasets introduce a level of complexity. The transition from dry to saturated conditions could potentially transpire anywhere within a sounding interval, thus complicating the precise identification of the saturation contact point.

Despite this limitation, for the purposes of this study, an assumption was adopted: the first sounding recorded immediately beneath the determined contact point is deemed to be in a saturated state. This supposition, albeit simplifying, assists in dealing with the intricacies derived from the constant depth intervals within our dataset.

Based on the (1:250,000) geological map (Healy et al. 1964), the clustering process successfully distinguished three hydrogeophysical classes but was unable to differentiate between weathered greywacke and weathered ignimbrite. Nonetheless, the interpretation of the SkyTEM data confirmed that the majority of the catchment consists of low permeability material, primarily deeply weathered ignimbrite transformed into clay. The presence of fractured Basaltic Andesite of the Kiwitahi Group was identified in the catchment headwaters, but the clustering approach failed to correctly identify greywacke in the lower basin to the northwest.

# **Appendix S2 Model development and calibration**

**Modelling platform selection**

For this study, the SWAT-MODFLOW-RT3D coupled model integrated through the QSWATMOD interface (Park et al. 2019) was selected. This choice was based on several key factors:

1. Computational efficiency: Unlike some fully integrated platforms that can have prohibitively long simulation times for regional-scale studies, SWAT-MODFLOW-RT3D maintains relatively short runtimes (around 7 to 30 minutes). This efficiency is crucial for performing multiple model runs necessary for uncertainty analysis and calibration.

2. Comprehensive simulation capabilities: The platform integrates surface water processes (SWAT), groundwater flow (MODFLOW), and reactive solute transport (RT3D). This combination allows for the simulation of both water quantity and quality, which is essential for our study of nitrogen flow paths.

3. Suitability for water quality analysis: The inclusion of RT3D enables detailed investigation of nutrient transport and transformation processes, making it particularly suitable for our focus on nitrate dynamics. While more advanced fully coupled models exist (e.g., ParFlow, HydroGeoSphere), SWAT-MODFLOW-RT3D offers a balance between process representation and computational demands that aligns well with our study objectives and available resources.

4. Previous applications: The platform has been successfully applied in similar contexts (Bailey et al., 2016; Wei et al., 2019; Wei & Bailey, 2021), providing a foundation of literature and methodology to build upon.

While this platform has limitations, such as simplified representation of some groundwater-surface water interactions compared to fully coupled models, it provides an appropriate tool for our specific research questions and scale of analysis. The choice of SWAT-MODFLOW-RT3D allows us to focus on data worth assessment and uncertainty analysis within a computationally feasible framework while still capturing the key hydrological and biogeochemical processes relevant to our study area.

It is worth noting that more recent developments in the SWAT family of models have also addressed groundwater representation. SWAT+ has been linked with MODFLOW, as demonstrated by Bailey et al. (2020). Furthermore, recent work by Yimer et al. (2023) has further improved the representation of groundwater-surface water interactions in SWAT+(gwflow) by incorporating a single layer representation of groundwater. While these advancements offer promising alternatives, the SWAT-MODFLOW-RT3D coupled model was chosen for this study due to its established track record in similar applications, its ability to handle multiple groundwater numerical layers and its balance of process representation and computational efficiency for our specific research objectives.

## **Model description**

As part of research investigating nutrient flow pathways in the Piako Headwaters catchment, a numerical flow and transport model was developed that linked SWAT, MODFLOW and RT3D (Clement & Johnson, 2012), using the QSWATMOD GIS interface. The model description is divided into surface water simulation, groundwater simulation and coupled simulation.

### **Surface water simulation**

Surface water quantity and quality were modelled using the SWAT 2012 platform (Arnold et al., 2013). The 108 km^2^ study area was divided into 21 sub-basins and river reaches (Figure B1a), using monitoring locations and topographical divides identified by LiDAR (Aerial Surveys, 2018. Airborne Laser Scan at 1 m resolution). Climate inputs were individually specified for each sub-basin. Computationally the sub-basins are broken into smaller hydrological response units (HRU) that are defined by soil and land cover classes. Soil spatial data was taken from the NZ fundamental soils layer and SMAP (Manaaki Whenua - Landcare Research 2019, Lilburne et al. 2012), with soil properties extracted from associated soil fact sheets and data collected by Manaaki Whenua Landcare Research for this study (Manaaki Whenua - Landcare Research 2019) and aggregated into a simple 1-layer implementation. Land cover spatial data was taken from the publicly available Land Cover Database version 5 (MFE 2020). Land cover was simplified into two classes: pastoral grazing and native forest. Land use properties were either left at default SWAT values while grazing statistics were taken from farm economic modelling conducted for the research programme (pers. Comm Journeaux, 2020): where plant consumption and effluent depositions for dairy grazing were specified as dry mass consumption ≈ 30 kg/d/ha and effluent dry mass deposition ≈ 12 kg/d/ha. These data were applied to the model via the SWAT land use management module (Arnold et al. 2013). No details were available for fertiliser application in the catchment, to account for this the effluent deposition mass was adjusted during calibration to compensate and to fit observed NO_3_-N concentrations in ground and surface water.

### Groundwater simulation

SWAT-MODFLOW-RT3D incorporates the MODFLOW-NWT (Niswonger et al. 2011) finite difference method to solve the general porous media groundwater flow equation. The model's spatial discretisation consists of 198 rows and 87 columns, forming square cells of 100 m by 100 m, and it is structured into two layers.

Two boundary condition configurations for the model domain were investigated. Initially, a closed boundary system was considered where water could only exit the domain via surface flow at Kiwitahi. The alternative explored involved setting the model domain's perimeter as general head boundary cells, where the heads were determined from interpolated groundwater levels derived from SkyTEM data, and conductance was informed by the hydraulic conductivity of the layers. However, since both configurations adequately matched the observed flow at Kiwitahi and minimal lateral flow was observed across the general head boundary cells, a closed boundary setup was ultimately selected.

Layer 1 of the MODFLOW model, represents the weathered ignimbrite and the shallow groundwater flow path (secondary interflow pathways) discussed in section 2.5. It extends hypothetically from 11 m below to 99 m above the LiDAR-derived land surface, totalling a uniform thickness of 110 m. While unusual, this approach was chosen to circumvent the model's coupling limitations that could otherwise induce erroneous transitions to confined groundwater conditions in instances of water table mounding. This issue is caused in SWAT-MODFLOW, by the lack of a mechanism to discharge groundwater to the land surface under such conditions (Guevara-Ochoa et al. 2020). Layer 2, with a uniform thickness of 89 m located beneath Layer 1, simulates the deeper groundwater system. This layer is crucial for modelling the more consistent baseflow contributions to streams and is where most monitoring and production wells are situated, and precluded the use of a more simplified 1-layer groundwater model.

In parallel, a Reactive Transport in 3-Dimensions (RT3D) model was adapted from the MODFLOW grid to simulate groundwater transport processes. Following the simplified RT3D reaction packages methodology by Wei and Bailey (2021), this component of our model aims to represent the transport dynamics, focussing primarily on denitrification. This implemented a simplified first-order kinetics reaction package for denitrification, with the first-order rate constant and Monod half-saturation constant included in the calibration process (ranges specified in Table S2-2). While porosity values were adjusted to reflect the variability across different hydro-stratigraphic units, the reactivity parameters were kept uniform across the model for computational efficiency. Dispersion coefficients for nitrate transport were fixed and estimated based on grid size and typical literature values. Longitudinal dispersivity was set to 2% of the characteristic length of a model cell (numerical experiments demonstrated that dispersivity could not be estimated based on the available observation data), while transverse and vertical dispersivity values were fixed at 10% of the longitudinal dispersivity, respectively.

### **Coupled simulation**

After independently developing the SWAT, MODFLOW and RT3D models, they were coupled using QSWATMOD, a QGIS-based tool designed for coupling SWAT and MODFLOW models, as outlined by Wei and Bailey (2021). This coupling uses the MODFLOW Rivers package to model water exchanges between surface and groundwater, adhering to the methodologies of Harbaugh et al. (2005). Due to the limitations of our datasets and the constraints of the modelling tools, streambed elevations were uniformly adjusted downward by 0.5m from LiDAR-derived ground surface elevations to approximate the streambed top. This adjustment was necessitated by a lack of cross-section data and the inherent limitation of infrared LiDAR, which reflects off the water surface, rendering direct bathymetric measurements unfeasible.

Field observations indicated that the 0.5 m offset might, in some cases, overestimate the actual depth of water in the streams and lead to a lower elevation estimate of the streambed.

In the context of this model, streambed conductance was adjusted to account for the absence of detailed bathymetric data, based on the MODFLOW Rivers package equation (Harbaugh, 2005):

| ￼ | **[S2-1]** |
| --- | --- |

and the river bottom is given by:

| 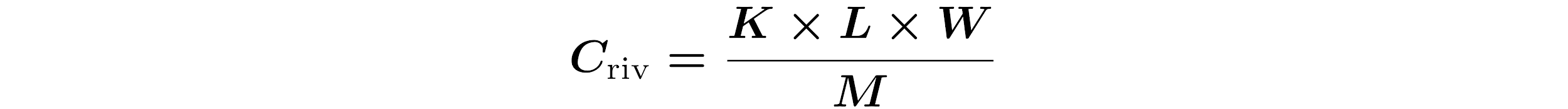 | **[S2-2]** |
| --- | --- |

where:

| ￼ | **[S2-3]** |
| --- | --- |

**Q** is the exchange flow between the river and the aquifer (positive for flow out of the river, negative for flow into the river).

**K** is the hydraulic conductivity of the streambed material.

**L** is the length of the river reach within a model cell.

**W** is the width of the river.

**M** is the thickness of the streambed.

**H**_riv_ is the head in the river.

**R**_bot_ is the elevation of the riverbed (river bottom).

These equations illustrate that the exchange is co-dependent on conductance and streambed elevation, allowing an error in one to be compensated by an adjustment in the other. Given the data limitations, we accepted that the streambed conductance value would serve as a compensatory factor for the imprecise knowledge of streambed elevation, as is usually the case in groundwater models using this conductance approach.

The hydrological response units of SWAT, which calculate the percolation of water out of the soil profile are disaggregated and mapped to the geographically corresponding MODFLOW grid cell during the coupling process, this allows groundwater recharge to be passed by SWAT to MODFLOW, (Bailey et al., 2016; Wei et al., 2019; Wei & Bailey, 2021). The temporal resolution of the model was set at a daily time step for all components.

The nitrate transport process in SWAT-MODFLOW-RT3D involves multiple steps across different model components. Initially, SWAT calculates the nitrate (NO_3_) mass in recharge water for each Hydrologic Response Unit (HRU), accounting for processes such as plant uptake, nitrification, and denitrification in the soil profile. This mass is then mapped to corresponding RT3D grid cells and converted to concentration values. RT3D subsequently solves the advection-dispersion-reaction equation for each grid cell (Equation [S2-4]), determining groundwater NO_3_ concentrations while considering processes like advection, dispersion, and denitrification in the aquifer. Finally, the model calculates mass exchange between the aquifer and streams. This is done by multiplying the cell groundwater NO_3_ concentration by the MODFLOW-simulated groundwater discharge rates for cells intersecting with stream networks. In areas where streams lose water to the aquifer, the model computes and subtracts the corresponding NO_3_ mass from the stream.

The nitrate lost from the soil profile after the process of nitrification and denitrification is given by the equation:


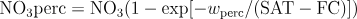
 **[S2-4]**

Where: NO_3_perc = amount of nitrate moved to the underlying layer (kg N/ha) w_perc_ = amount of water percolating to the underlying soil layer (mm H_2_O) SAT = saturated water content of the soil layer (mm H_2_O) FC = water content of the soil layer at field capacity (mm H_2_O)

Once the load leaves the soil profile and enters RT3D the transport and reaction is calculated by:

$$\frac{\partial C_{NO_{3}}}{\partial t}=-\frac{\partial}{\partial x_{i}}\left( \text{\textbackslash(}v_{i}C_{NO_{3}\text{\textbackslash)}} \right)+\frac{\partial}{\partial x_{i}}\left( \text{\textbackslash(}D_{ij}\frac{\partial C_{NO_{3}}}{\partial x_{j}}\text{\textbackslash)} \right)┤)$$

$\frac{+q_{s}}{\phi}C_{sNO_{3}}-k_{NO_{3}}C_{NO_{3}}\left( \text{\textbackslash(}\frac{C_{NO_{3}}}{K_{NO_{3}}+C_{NO_{3}}}\text{\textbackslash)} \right)$ **[S2-5]**

Where:

$\partial C_{NO_{3}}\text{/}\partial t$: The change in nitrate concentration with respect to time.

$v_{i}$: The velocity of the groundwater flow in the i-th direction.

$C_{NO_{3}}$: The concentration of nitrate.

$D_{ij}$ : The dispersion coefficient tensor, representing the spreading of nitrate due to both molecular diffusion and mechanical dispersion.

$q_{s}$: The source/sink term, which can account for additional inputs or losses of nitrate.

φ: The porosity of the medium, representing the fraction of the volume of voids over the total volume.

$C_{sNO_{3}}$: The concentration of nitrate in the source/sink term.

$k_{NO_{3}}$: The reaction rate coefficient for nitrate.

$K_{NO_{3}}$: The half-saturation constant for nitrate, representing the concentration at which the reaction rate is half of its maximum value.

### **Model Parameterisation**

In the present investigation, only a subset of the numerous SWAT variables underwent adjustment, while the remaining parameters retained their default values. The adjusted parameters were categorised into four groups corresponding to distinct geographic zones: the catchment's apex, the Piakonui, the Piakoiti, and the lowland region beneath the confluence (Figure S2-1a).

In the MODFLOW model, the hydro-stratigraphic units (3 per layer) are presumed to possess unique hydraulic properties, including horizontal conductivities (Kh), vertical anisotropies (Vani), and specific yields and storages (Sy, Ss) (Figure S2-1b). Furthermore, other parameters, such as streambed conductance, were implemented based on the four SWAT zones previously described. Porosity zonation follows the four SWAT zones.


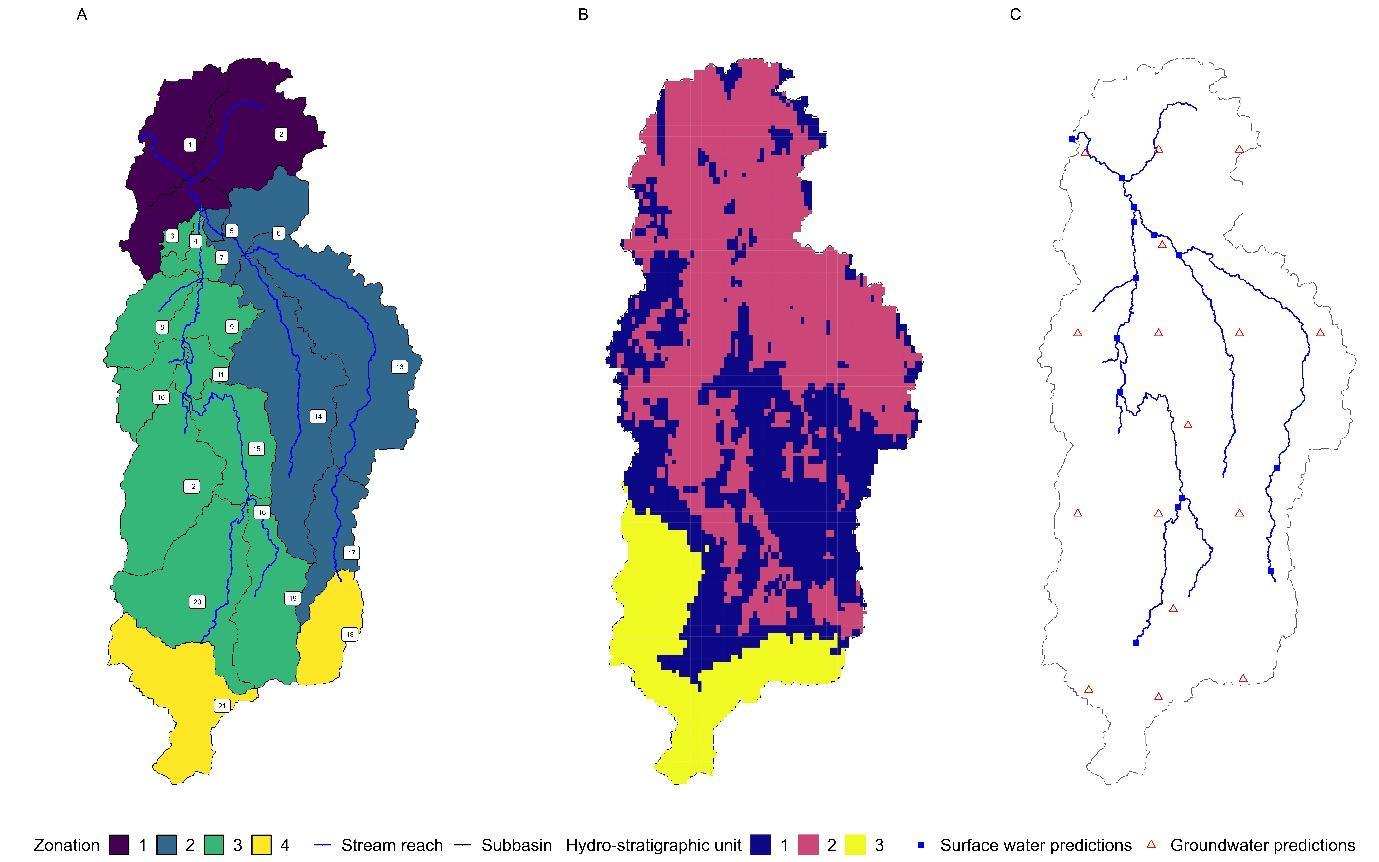


*Figure S1 Parameter zonation adopted (A: SWAT parameter zonation, B: MODFLOW parameter zonation)*

### **Model calibration**

Traditional approaches to model performance assessment often employ split-sample validation to assess model parameter robustness and simulation accuracy (Arsenault et al. 2018). However, Arsenault et al. (2018) challenge the efficacy of this method, instead demonstrating across multiple hydrological models and catchments that calibration using the full dataset consistently yields more robust parameter sets and superior model accuracy during independent testing periods. They advocate for the use of all available data in calibration to avoid the potential pitfalls of split-sampling, such as the rejection of viable parameter sets due to performance discrepancies between calibration and validation periods. In light of these recommendations and due to the limited period of data available in our study we adopt these recommendations, using the entirety of the available data for calibration.

To ensure a robust assessment of model performance beyond the normal objective function, we have modified traditional metrics, normalising them to account for observation errors.

Specifically, we use error-normalised versions of percent bias (PBIAS) (Gupta et al., 1999), R^2^ (Draper & Smith, 1998), RMSE (Willmott & Matsuura, 2005), and NSE (Nash, 1970).


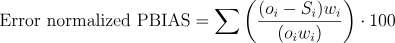
 **[S2-6]**


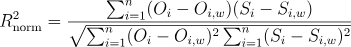
 **[S2-7]**


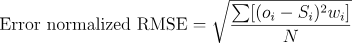
 **[S2-8]**


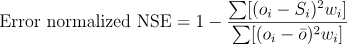
 **[S2-9]**

Where weight $w_{i}$is 1/observation error. Further, $i$ is the index of the number of observations **N**.

We note that this aligns closely with the PEST calibration approach thereby ensuring consistency between model calibration and performance assessment phases. By employing weights inversely proportional to observation errors, these metrics prioritise contributions from more reliable observations in the performance evaluation. This weighting mechanism ensures that observations with higher certainty exert a greater influence on the calculated metrics, reflecting their relative reliability and importance. For example, the error-normalized PBIAS offers a more nuanced measure of model bias by emphasising the discrepancies between observed and simulated values for which we have higher confidence, thereby providing an assessment that more accurately reflects the model's systematic deviations from reality. Similarly, the error-normalised RMSE and NSE adjust the contribution of each error term in the performance metrics, prioritising the minimisation of discrepancies for observations with lower associated errors. Incorporating error correction for uncertain observations in model performance evaluation is important, as it directly addresses the inherent inaccuracies and variabilities in the data that underpin model calibration processes. Ferro (2017), emphasises the challenges posed by observation errors in forecast verification and introduces error-corrected scoring rules to mitigate these issues. Observational uncertainties, which can stem from measurement inaccuracies, data processing anomalies, and representativeness issues, can significantly skew the evaluation of a model's predictive performance if not properly accounted for. Traditional performance metrics that neglect these uncertainties risk misrepresenting a model's capabilities, either by penalising the model for deviations from flawed observations or by failing to accurately assess its predictive accuracy.

A total of 14 observation groups were used during model calibration. The basis for these observation groups consisted of Markov Chain Monte Carlo derived flow estimates from river stage (see Appendix S4 for discussion on inclusion and associated uncertainties in predictions), monthly grab samples for NO_3_-N concentrations, continuously recorded surface water nitrate concentrations at two locations, spot groundwater levels at 29 locations, continuously recorded groundwater levels at two locations and 3746 SkyTEM derived groundwater levels (Figure 1 of main text). As is common practice when using PEST to calibrate groundwater models, the observation weights for the objective function used during calibration were determined using the inverse of the measurement error for each observation. The observations were then grouped (Table S2-1) by their corresponding types and locations, as appropriate, and each group was given equal weight.

Due to the long simulation times of the coupled SWAT-MODFLOW-RT3D model (ranging from 10 to 60 minutes for a 7-year simulation) and the extended period required for deep groundwater (numerical layer 2) to reach equilibrium (several decades), the following sequential calibration strategy was implemented:

1. A daily, 21-year simulation of the SWAT model was calibrated using PEST to estimate initial SWAT parameter values for the coupled model, as well as to estimate the parameters that are sensitive to groundwater recharge. A total of 60 parameters (as shown in Table 2 with grey shading) were adjusted to minimise the objective function, which consisted of 18,308 observations based on the observation groups shown in Table 3.
2. After the optimisation of SWAT parameters, the SWAT model was then coupled to the MODFLOW model and a 21 year forward run was conducted using the parameters generated in the previous step. The purpose of this step was to generate recharge inputs for an uncoupled MODFLOW model used to generate initial groundwater heads for each simulation.
3. Next, the coupled model was calibrated for 79 parameters. Parameters were mapped to both an uncoupled MODFLOW model and the coupled model. The uncoupled MODFLOW model, using recharge from step 2, was run to reach the equilibrium state before each coupled simulation, with the heads from the final timestep passed as initial conditions to the coupled model. SWAT recharge parameters were fixed to the values obtained from step 1. The MODFLOW model was run at a yearly timestep for 68 years, recycling the 21 years of SWAT recharge, and then for 3 years at a monthly timestep. This simulation length was chosen by trial and error to reflect the very low permeability in the aquifer (represented by numerical layer 2). Following a trial and error approach the initial conditions for RT3D were fixed at 0, reflective of the generally very low concentrations observed in the deeper aquifer. The coupled model, including RT3D, was run for 7 years with a 1-year warm-up period for SWAT. The model objective function used a total of 22973 observations during the simulation period. Observations from step 1, available during the reduced simulation period, were supplemented with the addition of groundwater head and nitrate measurements and 11194 targets of no groundwater heads above the land surface.

During initial calibration, it was observed that due to the dynamic nature of NO_3_-N concentrations in the streams, BeoPEST tended to produce unsatisfactory results. This was due to the objective function metric (summed mean square error) meeting the mean value but not capturing the dynamic nature of the nitrate concentrations in surface water. To obtain a better fit for all the NO_3_-N data, statistical metrics for nitrate observations (mean, maximum and minimum recorded values) were supplemented into the objective function. Additionally, NSE at the catchment outlet at Kiwitahi was supplemented into the objective function to better represent flow dynamics.

Table S2-1 Calibration target groups

| **Observation group** | **Initial SWAT model** | **SWAT-MODFLOW-RT3D** |
| --- | --- | --- |
| **Log flow at Kiwitahi** | X | X |
| **Cumulative discharge at Kiwitahi** | X | X |
| **Monthly grab-sample NO3-N concentrations at Kiwitahi** | X | X |
| **Monthly grab-sample NO3-N concentrations at Piakonui Pt-Road** | X | X |
| **Log Flow at Piakoiti above the confluence and at the upper reaches** | X | X |
| **Log Flow at Piakonui above the confluence and at the upper reaches** | X | X |
| **Continuous NO3-N concentrations at Piakonui above the confluence with Piakoiti** | X | X |
| **Continuous NO3-N concentrations at Piakoiti above the confluence with Piakonui** | X | X |
| **Continuous Groundwater levels** |  | X |
| **SkyTEM-derived groundwater levels** |  | X |
| **Spot groundwater levels** |  | X |
| **Groundwater NO3-N concentrations** |  | X |
| **No cell flooding** |  | X |
| **NSE at Kiwitahi** | X | X |
| **Mean, minimum and maximum NO3-N at Kiwitahi** |  | X |
| **Mean, minimum and maximum NO3-N at Piakonui above the confluence with Piakoiti** |  | X |
| **Mean, minimum and maximum NO3-N at Piakoiti above the confluence with Piakonui** |  | X |

## **Model calibration results**

### **Calibrated parameter values**

The BEOPEST calibrated set of parameters of the calibrated model is presented in Table S2-2. Following model calibration, parameter identifiability has been assessed and the model performance has been assessed using error normalised statistical metrics for stream flow, in-stream nitrate concentrations, groundwater levels and groundwater nitrate concentrations.

*Table S2-2 Calibrated parameters*

| *Name* | *# of parameters* | *Input Range* | *Calibrated value range* |
| --- | --- | --- | --- |
| *K_h_ layer1 (m/d)* | 3 | 1 to 150 | 41.42, 76.42 and 139 |
| *K_h_ layer2 (m/d)* | 3 | 0.0001 to 20 | 1x10^-4^, 9x10^-4^ to 0.0403 |
| *S_y_ layer 1* | 3 | 0.0001 to 0.2 | 1.3x10^-4^,1.5x10^-4^ and 1.5x10^-4^ |
| *S_y_ layer 2* | 3 | 0.005 to 0.4 | 5.6x10^-3^, 7.34x10^-3^ and 0.315 |
| *V_ani_ layer 1* | 3 | 1 to 20 | 1, 5.64 and 13 |
| *V_ani_ layer 2* | 3 | 0.1 to 20 | 0.214, 1 and 2.27 |
| *MODFLOW river conductance (m/d) (vertical conductance times length of stream segment in MODFLOW cell time wides of stream segment)* | 4 | 0.05 to 20000 | 40, 53, 453 and 6950 |
| *Porosity* | 4 | 0.01 to 0.5 | 0.01, 0.016, 0.0398, 0.0341 and 0.0438 |
| *First-order rate constant for denitrification (1/T)* | 1 | 0.0001 to 10 | 4.88E-02 |
| *Monod half-saturation term for denitrification* | 1 | 0.1 to 600 | 34.745 |
| *Baseflow alpha factor (d^-1^) (SWAT only)* | 4 | 0.05 to 0.999 | 0.09 to 0.547 |
| *Groundwater delay (d)* | 3 | 0.05 to 100 | 0.2 to 27.4 |
| *Baseflow alpha factor for bank storage (d)* | 4 | 0.1 to 1 | 0.56 to 1 |
| *SWAT channel conductance cm/hr (SWAT only)* | 4 | 0.1 to 10000 | NA |
| *Manning's "n" for main channel* | 4 | 0.025 to 0.1 | 0.028 to 0.05 |
| *Manning's "n" value for overland flow* | 4 | 0.025 to 0.1 | 0.03 to 0.0445 |
| *Average slope length for sheet flow (m)* | 4 | 0.1 to 200 | 2.4 to 104 |
| *Slope length for lateral subsurface flow (m)* | 4 | 0.1 to 200 | 27 to 199 |
| *Soil available water content (mm)* | 2 | 60 to 250 | 200 to 243 |
| *Soil k_sat_ (cm/hr)* | 3 | 2 to 180 | 2.77, 105 and 128 |
| *Surface runoff lag coefficient* | 1 | 1 to 40 | 16.6 |
| *Half-life of nitrate in the shallow aquifer (d) (SWAT only)* | 1 | 1 to 1000 | 16.1 |
| *Nitrate percolation coefficient* | 1 | 0.001 to 1 | 0.00583 |
| *Denitrification exponential rate coefficient* | 1 | 0.01 to 0.99 | 0.217 |
| *Denitrification threshold water content* | 1 | 0.01 to 2 | 0.9507 |
| *Secondary channel Manning’s n* | 4 | 0.025 to 0.1 | 0.025 to 0.0894 |
| *Secondary channel conductance cm/hr* | 4 | 1E^-9^ to 150 | 5.1 to 6.7 |
| *Pasture dry mass consumption (kg/ha)* | 4 | 20 to 40 | 27 to 40 |
| *Effluent dry mass (kg/ha)* | 4 | 8 to 15 | 10 to 12 |
| *Canopy interception pasture (mm)* | 1 | 0.1 to 4 | 1.43 |
| *Canopy interception native forest (mm)* | 1 | 0.5 to 10 | 0.86 |

## Parameter identifiability

Parameter identifiability assessment evaluates how much the uncertainty in a parameter is reduced through the calibration process. It indicates which parameters are most influenced by the calibration data and which have the greatest impact on model outputs. The results of parameter identifiability are given in Figure S2, which illustrates the relative reduction in uncertainty following calibration for each parameter. A value close to unity indicates that the parameter values were not informed by the calibration dataset. While values close to zero indicate that the parameter value was highly informed by the calibration process.


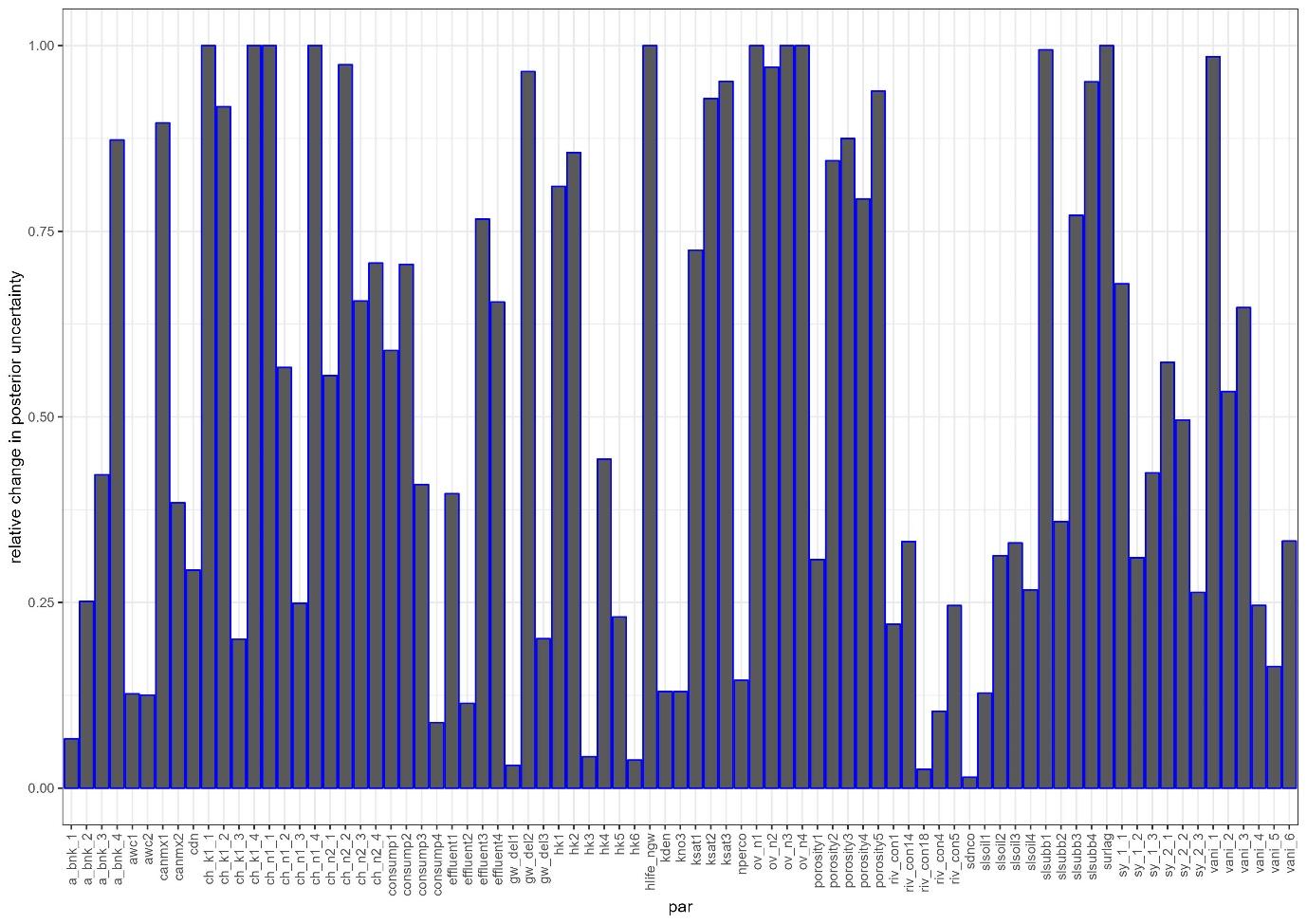


*Figure S2 relative change in parameter uncertainty following calibration (smaller values represent greatest reduction)*

From Figure S2 it can be seen that the uncertainty of the groundwater (MODFLOW) parameters and those SWAT parameters which control recharge, have the greatest reductions in uncertainty. This is understandable from the perspective of model behaviour and the choice of model timesteps. The objective function of the model (**Φ**) is composed of surface water flow and nitrate concentration targets, in addition to groundwater levels and nitrate concentration targets. Parameters such as groundwater hydraulic conductivity (K_h_), specific yield (S_y_) (and storage S_s_), and stream-bed conductance (C) affect estimates of all these targets. For instance, the hydraulic conductivity, specific yield, and specific storage control groundwater heads, which also determines the position of the groundwater table in relation to the stream bed, thus controlling whether the stream is gaining or losing water. Similarly, the stream-bed conductance parameter affects the rate of exchange between the stream and groundwater. In contrast, few SWAT parameters have such wide-ranging impacts on the model outputs. The parameters that do, such as those that control groundwater recharge and nitrate load generation (available water content, soil saturated conductivity, slope length, effluent load, dry matter consumed), have been shown to be highly identifiable in assessments of uncertainty reductions. Conversely, parameters that affect limited parts of the model outputs or that would have impacts at sub-daily time steps, such as Manning's roughness, are less identifiable.

### **Stream flow and nitrate concentrations**

Table S2 outlines the model's performance against the calibration datasets. It is important to note that flows were log-transformed during the calibration process to emphasise the study's focus on groundwater and surface water interactions. This approach is used because non-transformed data in the calibration objective function could bias the calibration metrics towards high flows, potentially leading to suboptimal model performance during periods of low flow. Low-flow periods are particularly representative of groundwater and surface water interactions, especially in gaining reaches such as those observed in the Piako Headwaters catchment. This methodological choice ensures that our model accurately captures the critical dynamics of these interactions across the full range of flow conditions. For the majority of stream flows, the error-normalised NSE values are near unity, signifying a strong alignment between model predictions and observed data, given the rated flow measurement uncertainties. However, lower error-normalised NSE and R^2^ and higher RMSE values for site PKT5 indicate subpar performance at this location, as further depicted in Figure B2. This points to the model's challenges in accurately representing the upper Piakonui area's conditions, possibly due to issues related to boundary conditions or water tables in steep terrains.

Figure S3 also highlights the measurement uncertainty associated with the flow data, as shown by the rated flow interval. The MCMC-derived flow ratings (Appendix S4) showed that the 95% confidence interval for mean flow in Kiwitahi between 1999 and 2022 exhibits a ±25% to ±85% variance. During the period of coupled simulations, this interval narrows to ±25% to ±41%. For the additional stage recorder sites instrumented for this study, the variance in the 95% confidence interval extends from ±7% to as high as ±115%. The increased uncertainty at these sites is largely due to limited gaugings and unstable cross-sections at these temporary monitoring stations.

Regarding surface water NO_3_-N concentrations, normalised PBIAS values at three of the four examined sites are adequate. However, scrutiny of Figure S2-3 suggests limitations in the utility of the normalised PBIAS metric for evaluating model performance, particularly at the PKN5 site. Here, as at the other site, model outputs are within the observation error, indicating that normalised PBIAS is not necessarily a robust metric in this context. Figures S2-2 and S2-3 provide a visual representation of the model's fit to the observed data and associated uncertainties.

*Table S2-3 Surface water calibration statistics*

| Observation group | Mean observation error | Error Normalised  RMSE (unit of measurement) | R^2^ | Error Normalised R^2^ | NSE | Error Normalised NSE | Pbias (percentage) | Error NormaliseD Pbias (percentage) |
| --- | --- | --- | --- | --- | --- | --- | --- | --- |
| Kiwitahi Flow (m^3)^ | 0.44 | 1.11 | 0.61 | 0.74 | 0.6 | 0.99 | - | - |
| Kiwitahi NO_3_-N mg/L | <0.3 mg/L = 0.5  >0.3 mg/L = 12%  Mean = 0.26 | - | 0.69 | - |  | - | -50 | -3.6 |
| PKN1  Flow (m^3^) | 0.20 | 0.83 | 0.8 | 0.64 | 0.48 | 0.95 |  | - |
| PKN1  NO_3_-N mg/L | <0.5 mg/L = 0.5  >0.5 mg/L = 5%  Mean = 0.29 | NA | 0.52 | NA |  | NA | 22.5 | -13.2 |
| PKNT2  Flow (m^3)^ | 0.05 | 0.22 | 0.55 | 0.62 | 0.51 | 0.95 |  | - |
| PKN4  Flow (m^3)^ | 0.04 | 0.90 | 0.65 | 0.08 | 0.59 | 0.50 |  | - |
| PKN5  Flow (m^3)^ | 0.02 | 0.58 | 0.44 | 0.32 | -0.05 | 0.85 |  | - |
| PkN5  NO_3_-N mg/L | <0.3 mg/L = 0.5  >0.3 mg/L = 12%  Mean = 0.26 | - | 0.34 | - |  | - | -62.1 | 69 |
| Pkt1  Flow (m^3)^ | 0.12 | 1.07 | 0.83 | 0.63 | 0.24 | 0.90 |  | - |
| Pkt1  NO_3_-N mg/L | <0.5 mg/L = 0.5  >0.5 mg/L = 5%  Mean = 0.29 | NA | 0.67 | NA |  | NA | 29 | 6.5 |
| PKT5  Flow (m^3)^ | 0.01 | 0.33 | 0.58 | 0.63 | -0.72 | 0.31 |  | - |


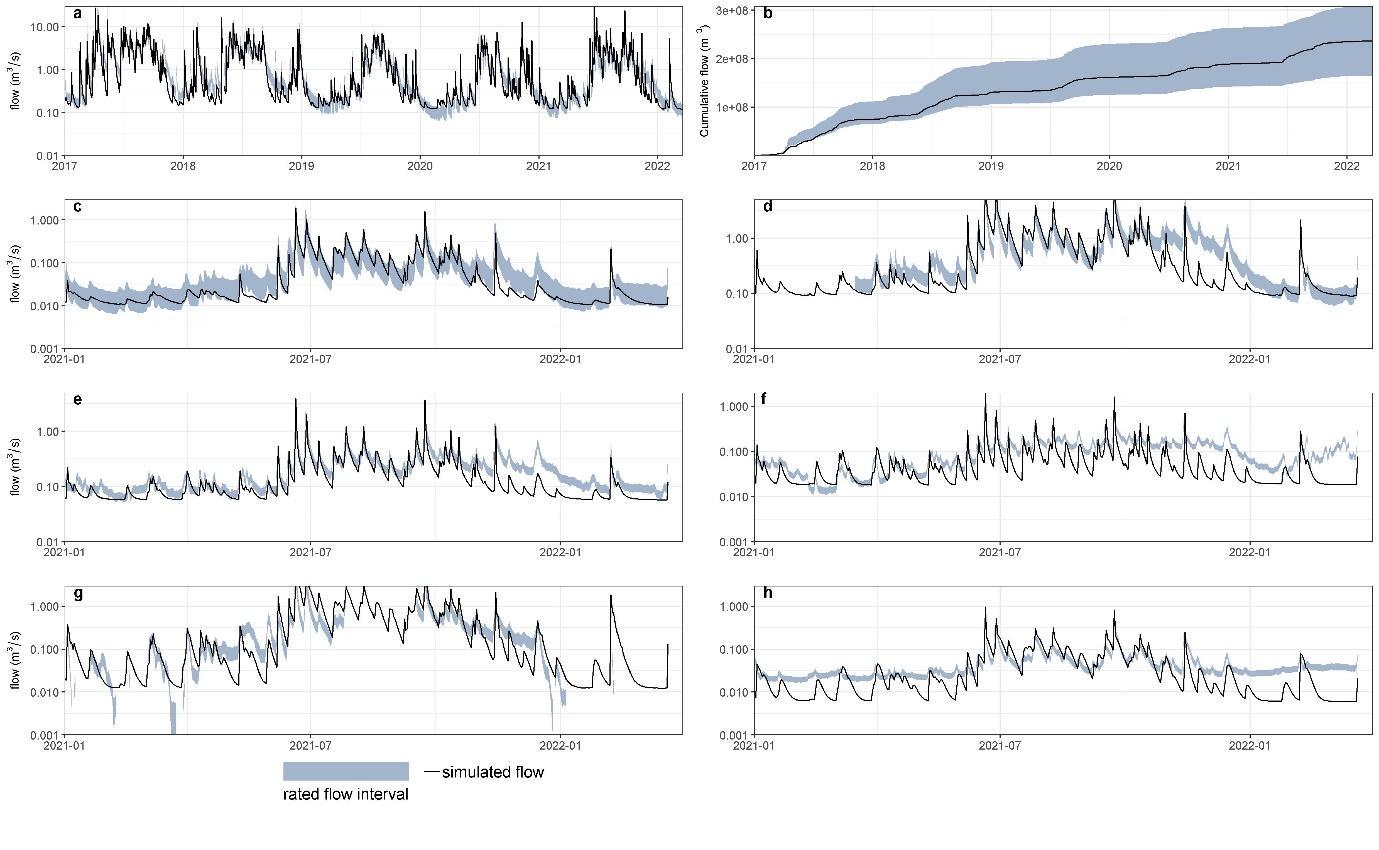


*Figure S3 Flow @ a. Kiwitahi Recorder, b. Cumulative discharge volume at Kiwitahi, c. PKNT2, d. PKN1, e. PKN4, f. PKN5, g. PKT1, h. PKT5.*


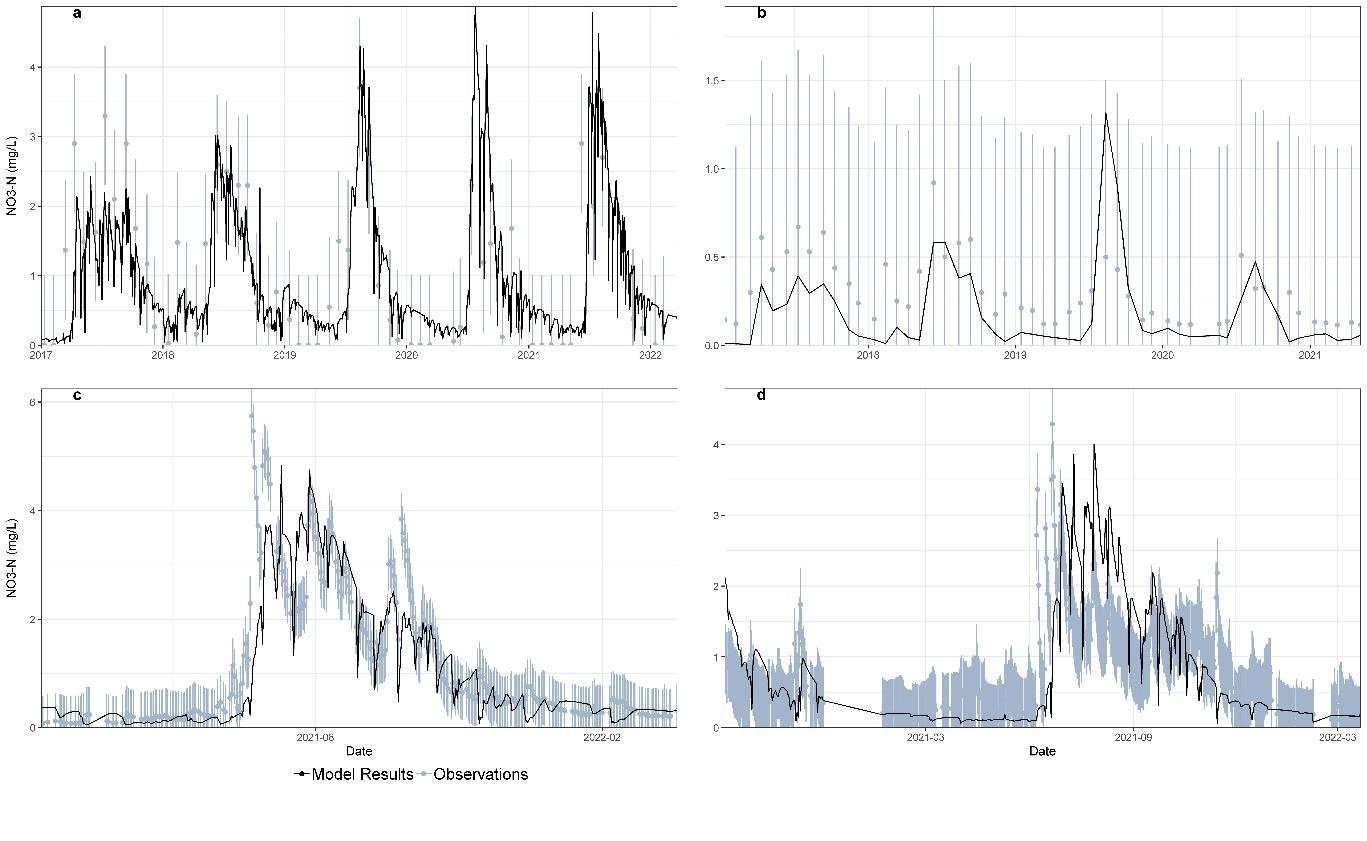


*Figure S4 Nitrate concentrations @ a. Kiwitahi, b. PKN-5, c. PKT-1, d. PKN-1*

### **Groundwater levels and nitrate concentrations**

Table S2-4 shows that the model performs well in predicting SkyTEM Derived Groundwater levels (STDGWL), evidenced by an R² value exceeding 99% and an error normalised RMSE below 5.18 meters (Figure S2-4 a). Conversely, the model performs relatively poorly against one-off groundwater level measurements, as shown by a higher error-normalized RMSE of 9.7 m, indicating limitations in scenarios with sparse data points.

Notably, the model's performance improves substantially for transient water level observations, achieving a Normalised RMSE of merely 0.2 meters. Despite some inaccuracies in capturing seasonal fluctuations (Figure S2-4 b & c), the overall performance for these transient sites is good.

The inability to capture the range of seasonal groundwater fluctuations is one possible cause of the poor performance against spot groundwater levels records; this may suggest the model is biased to the SkyTEM-derived water levels, though we note the uncertainty in the SkyTEM estimates is greater than the seasonal fluctuations. This would imply the model is structurally biased and either too coarse in horizontal grid resolution or vertical discretisation.

Regarding groundwater nitrate concentrations, the model's performance is moderate, with an R² value of 0.28 and an error-normalised RMSE of 1.48 mg/L. This performance can likely be attributed to the limited number of observational data points (12 grab samples) and their low influence on the model's objective function. Potential structural errors in the model may also be a contributing factor.

*Table S2-4 Groundwater calibration statistics*

| Observation group | Mean observation error | Error Normalised RMSE | Error Normalised R^2^ |
| --- | --- | --- | --- |
| STDGWL (SkyTEM) (m) | 3.77 | 5.18 | 0.99 |
| Spot GW levels (m) | 3.83 | 9.68 | 0.87 |
| LAL wells (m) | 1.00 | 0.20 | 1.00 |
| GW Nitrate concentration (mg/L) | 0.4 (30%) | 1.48 | 0.28 |


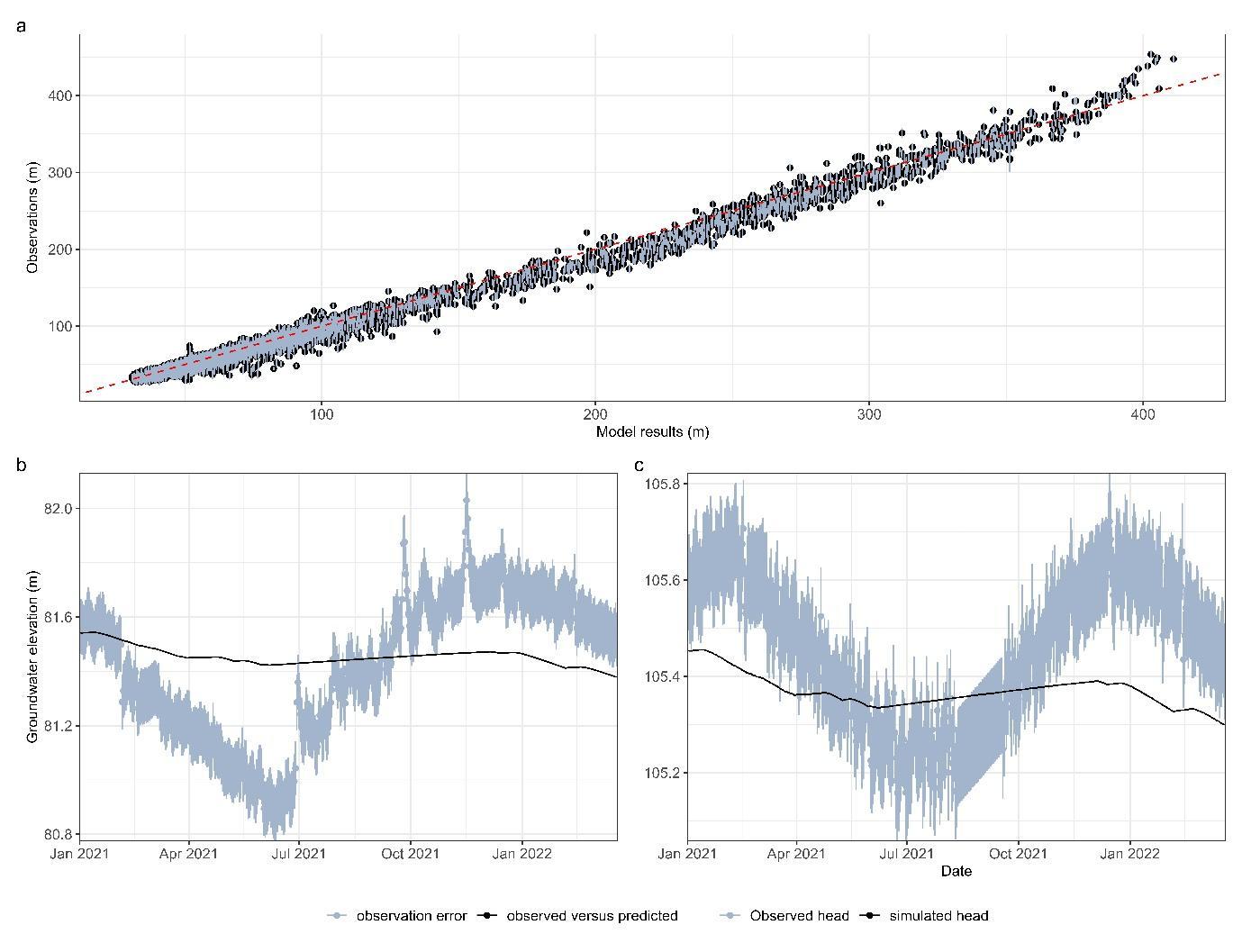


*Figure S2-4 STDGWL estimated vs simulated head (a), modelled head at LAL wells b. L1 and c. L2*

Despite a successful calibration indicated by a high R² value and low error-normalized RMSE, the model exhibits several limitations. It performs less reliably in areas of high elevation and for spot groundwater levels. Additionally, there is a consistent overprediction of head elevation, particularly in the southern domain. These shortcomings are likely due to structural errors in the model, which might stem from the use of SkyTEM clusters and a simplified two-layer approach. Previous research by Christensen et al. (2017) warns that SkyTEM-derived structures can introduce prediction errors.

Improving model complexity, either through additional layers or more variable hydraulic conductivity fields, may enhance performance. Challenges also arise in simulating groundwater levels in steep river valleys, possibly due to slope, grid resolution, and the absence of a feedback mechanism between groundwater, soil moisture, and stream flow in SWAT-MODFLOW (Guevara-Ochoa et al., 2020). Despite these limitations, the model remains largely effective in predicting groundwater levels, surface water flow, and nutrient transport.

# **Appendix S3 SkyTEM Aerial Geophysics Derived Groundwater Levels**

The methodology calculates the mean resistivity from individual soundings, identifying the depth where a notable drop in resistivity is observed. This change is assumed to signify the transition from saturated to unsaturated conditions, helping pinpoint the water table's probable location. This approach aligns with Vang et al.'s (2023) approach for NMR data. Estimates were made for each sounding and then resampled via k-nearest neighbours to adapt them to the model grid. Spot checks against eight available wells showed that six fell within SkyTEM's estimated confidence limits. The two outliers are located in the model's second layer and are not indicative of the groundwater table. Despite an often greater than 5 m uncertainty due to the water table's depth, the approach improves our understanding of potential water table elevations and their associated uncertainties, while also providing a snapshot of conditions on the SkyTEM flight days (18-19/2/2018).

# **Appendix S4 Flow target derivation**

The raw flow data collected for this study was in the form of stage height and cross-sectional shape and area. To derive an estimate of flow for incorporation into the modelling, the Power law model with constant variance was applied in a Bayesian hierarchical implementation using the Markov Chain Monte Carlo (MCMC) simulation tool BDRC (Hrafnkelsson et al. 2021).

$Q\left( h \right)={a\left( h-c \right)}^{b}$ **(S4-1)**

Where **Q** is river discharge, **h** is river stage and **a**, **b** and **c** are variables adjusted to fit the data.

The BDRC approach enables robust incorporation of a range of flow predictions possible for the stage data and directly recognises the flow prediction uncertainty.

As the previously derived rated flow for the Kiwitahi Recorder site also lacked an assessment of uncertainty, the rating curves were re-derived using the same ensemble approach to ensure consistency in the simulations.

The MCMC sampling approach produces modelled flow predictions at the 95^th^ percent confidence interval, e.g., 2.5^th^ percentile, median, and 97.5^th^ percentile. To set observation targets and weights in PEST, the BDRC results are assumed to be Gaussian (an assumption of the BDRC approach), and the rated flow target is set to the mean flow prediction. The weight is specified as 1/error, where the error is assumed to be half the 95^th^ % confidence interval:

$\frac{97.5thpercentile-2.5thpercentile}{2}$ .

# **Appendix S5 Relative ranking of data observation types**

*Table S5-1 Relative ranking by fall in uncertainty variance accrued with the inclusion of each observation group as the sole member of the calibration dataset.*

| Prediction | Groundwater head | | | Groundwater nitrate | | | Surface water discharge | | | Surface water nitrate | | |
| --- | --- | --- | --- | --- | --- | --- | --- | --- | --- | --- | --- | --- |
| Percentile | 5 | 50 | 95 | 5 | 50 | 95 | 5 | 50 | 95 | 5 | 50 | 95 |
| in catchment flow | 3 | 3 | 3 | 3 | 4 | 4 | 2 | **1** | 2 | 4 | 4 | 4 |
| catchment outlet flow | 7 | 7 | 7 | 7 | 8 | 8 | 6 | 3 | 3 | 6 | 6 | 6 |
| spot gw levels | 2 | 2 | 2 | 5 | 6 | 6 | 7 | 7 | 7 | 7 | 8 | 8 |
| continuous gw levels | 4 | 4 | 4 | 6 | 7 | 7 | 5 | 6 | 6 | 8 | 7 | 7 |
| gw nitrate | 8 | 8 | 8 | 8 | 3 | 3 | 8 | 8 | 8 | 3 | 3 | 3 |
| SkyTEM gw levels | **1** | **1** | **1** | 4 | 5 | 5 | **1** | 5 | 5 | 5 | 5 | 5 |
| grab sample nitrate | 6 | 6 | 6 | 2 | 2 | 2 | 4 | 4 | 4 | 2 | 2 | 2 |
| continuous nitrate | 5 | 5 | 5 | **1** | **1** | **1** | 3 | 2 | **1** | **1** | **1** | **1** |

*Table S5-2 Ranking of observation type data worth with respect to rise in uncertainty following observation omission*

| Prediction | Groundwater head | | | Groundwater nitrate | | | Surface water discharge | | | Surface water nitrate | | |
| --- | --- | --- | --- | --- | --- | --- | --- | --- | --- | --- | --- | --- |
| Percentile | **5** | **50** | **95** | **5** | **50** | **95** | **5** | **50** | **95** | **5** | **50** | **95** |
| in catchment flow | 5 | 5 | 5 | 5 | 5 | 5 | 3 | 2 | 3 | 4 | 4 | 4 |
| catchment outlet flow | 7 | 7 | 7 | 7 | 7 | 7 | 6 | 5 | 5 | 6 | 5 | 6 |
| spot gw levels | 6 | 6 | 6 | 8 | 8 | 8 | 7 | 7 | 7 | 8 | 8 | 7 |
| continuous gw levels | 2 | 2 | 2 | 6 | 6 | 6 | 5 | 6 | 6 | 7 | 6 | 5 |
| gw nitrate | 8 | 8 | 8 | 4 | 2 | 2 | 8 | 8 | 8 | 5 | 7 | 8 |
| SkyTEM gw levels | **1** | **1** | **1** | **1** | **1** | **1** | **1** | 3 | **1** | 3 | 3 | 3 |
| grab sample nitrate | 4 | 4 | 4 | 3 | 4 | 4 | 4 | 4 | 4 | 2 | 2 | 2 |
| continuous nitrate | 3 | 3 | 3 | 2 | 3 | 3 | 2 | **1** | 2 | **1** | **1** | **1** |
